# DStat: A Versatile, Open-Source Potentiostat for Electroanalysis and Integration

**DOI:** 10.1371/journal.pone.0140349

**Published:** 2015-10-28

**Authors:** Michael D. M. Dryden, Aaron R. Wheeler

**Affiliations:** 1 Department of Chemistry, University of Toronto, Toronto, ON, Canada; 2 Institute for Biomaterials and Biomedical Engineering, University of Toronto, Toronto, ON, Canada; 3 Donnelly Centre for Cellular and Biomolecular Research, 160 College Street, Toronto, ON, Canada; University of Illinois at Chicago, UNITED STATES

## Abstract

Most electroanalytical techniques require the precise control of the potentials in an electrochemical cell using a potentiostat. Commercial potentiostats function as “black boxes,” giving limited information about their circuitry and behaviour which can make development of new measurement techniques and integration with other instruments challenging. Recently, a number of lab-built potentiostats have emerged with various design goals including low manufacturing cost and field-portability, but notably lacking is an accessible potentiostat designed for general lab use, focusing on measurement quality combined with ease of use and versatility. To fill this gap, we introduce DStat (http://microfluidics.utoronto.ca/dstat), an open-source, general-purpose potentiostat for use alone or integrated with other instruments. DStat offers picoampere current measurement capabilities, a compact USB-powered design, and user-friendly cross-platform software. DStat is easy and inexpensive to build, may be modified freely, and achieves good performance at low current levels not accessible to other lab-built instruments. In head-to-head tests, DStat’s voltammetric measurements are much more sensitive than those of “CheapStat” (a popular open-source potentiostat described previously), and are comparable to those of a compact commercial “black box” potentiostat. Likewise, in head-to-head tests, DStat’s potentiometric precision is similar to that of a commercial pH meter. Most importantly, the versatility of DStat was demonstrated through integration with the open-source DropBot digital microfluidics platform. In sum, we propose that DStat is a valuable contribution to the “open source” movement in analytical science, which is allowing users to adapt their tools to their experiments rather than alter their experiments to be compatible with their tools.

## Introduction

Electrochemistry is an important topic of modern chemical research, spanning analytical, inorganic, organic, physical chemistry, and beyond. An abundance of electroanalytical techniques have been developed and most require the precise control of potentials in an electrochemical cell. Naïvely, one might suggest controlling the applied potential with a simple circuit such as a voltage divider in series with a battery. This strategy fails, however, as current passing through the cell causes a potential drop across the source’s impedance (and there are practical limits to how small the source’s output impedance can be made), resulting in considerable error even for low current flow. Further, in many electrochemical experiments, the cell current varies significantly with time, causing drastic changes in the apparent potential. These challenges led to the development of the potentiostat in the 1940s. [1]

Modern potentiostats provide a reliable means of controlling the potential of a working electrode in an electrochemical cell, relying on feedback to accurately maintain the cell potential regardless of changes in cell impedance during the measurement. Potentiostats can be purchased as rack-mount units for corrosion and energy research, bench-top instruments for analytical use, and even pocket-sized devices for blood glucose measurement. Unfortunately, most commercial potentiostats operate as “black boxes” with limited information available to users about their circuitry and behaviour. As noted by Bard and Faulkner, [2] this imposes significant constraints on researchers who are developing new measurement techniques and applications.

The “black box” potentiostat conundrum has recently spawned a cottage industry of home-made potentiostats that can be modified as needed for varying applications. These circuits largely fall into four classes: (1) tiny instruments intended for implantation or wearable use, [3–7] (2) inexpensive instruments designed to feature extreme cost savings or field-portability [8–12] (at the expense of performance), (3) multiplexed instruments designed for specialized applications involving arrays of electrodes, [13–15] or (4) bench-scale instruments constructed around “virtual instrument” frameworks [15–17] (which are useful for prototyping, but are expensive and lack robustness and portability). We applaud the “open-source” philosophy that has driven the development of lab-built potentiostats, which is part of a broader movement in analytical science that is allowing users to adapt their tools to suit their experiments rather than altering their experiments to suit their tools. [18] But none of the previously reported circuits [3–17] are a perfect match for general experimental use—that is, there are no lab-built potentiostats that match all of the characteristics that are associated with commercial potentiostats (high precision, low noise, compatibility with low current measurements, robustness, ease of use, and portability). Further, while considerable detail has been reported for many of the homemade potentiostats described previously [4, 10, 12], only two of them are truly open source [8, 9]—with explicit rights granted and encouragement given to any user who wishes to construct and modify the original design. There is thus great need for an open-source potentiostat intended for use in research, featuring picoampere-level measurement capabilities, compact and robust form-factor, and intuitive cross-platform software.

In recognition of the need described above, we introduce DStat, an open-source potentiostat designed for high-performance laboratory experimentation. All salient details about DStat, including schematics, parts lists, control software, and assembly instructions, are available at http://microfluidics.utoronto.ca/dstat. The open nature of DStat makes it attractive when compared to commercial instruments; all aspects of DStat’s operation are disclosed and fully modifiable, facilitating adaptation to experiments for which it was not initially designed as well as integration with other instruments. Here we describe the design of DStat, and compare its performance with that of (a) a “black box” commercial potentiostat (EmStat 1, PalmSens BV, Utrecht, NL), (b) a popular open-source potentiostat reported previously, the “CheapStat,” [8] and (c) a commercial benchtop pH meter (Accumet AR50 benchtop meter, Thermo Fisher Scientific, Waltham MA, USA).

## Materials and Methods

### Design

**Fig 1:**
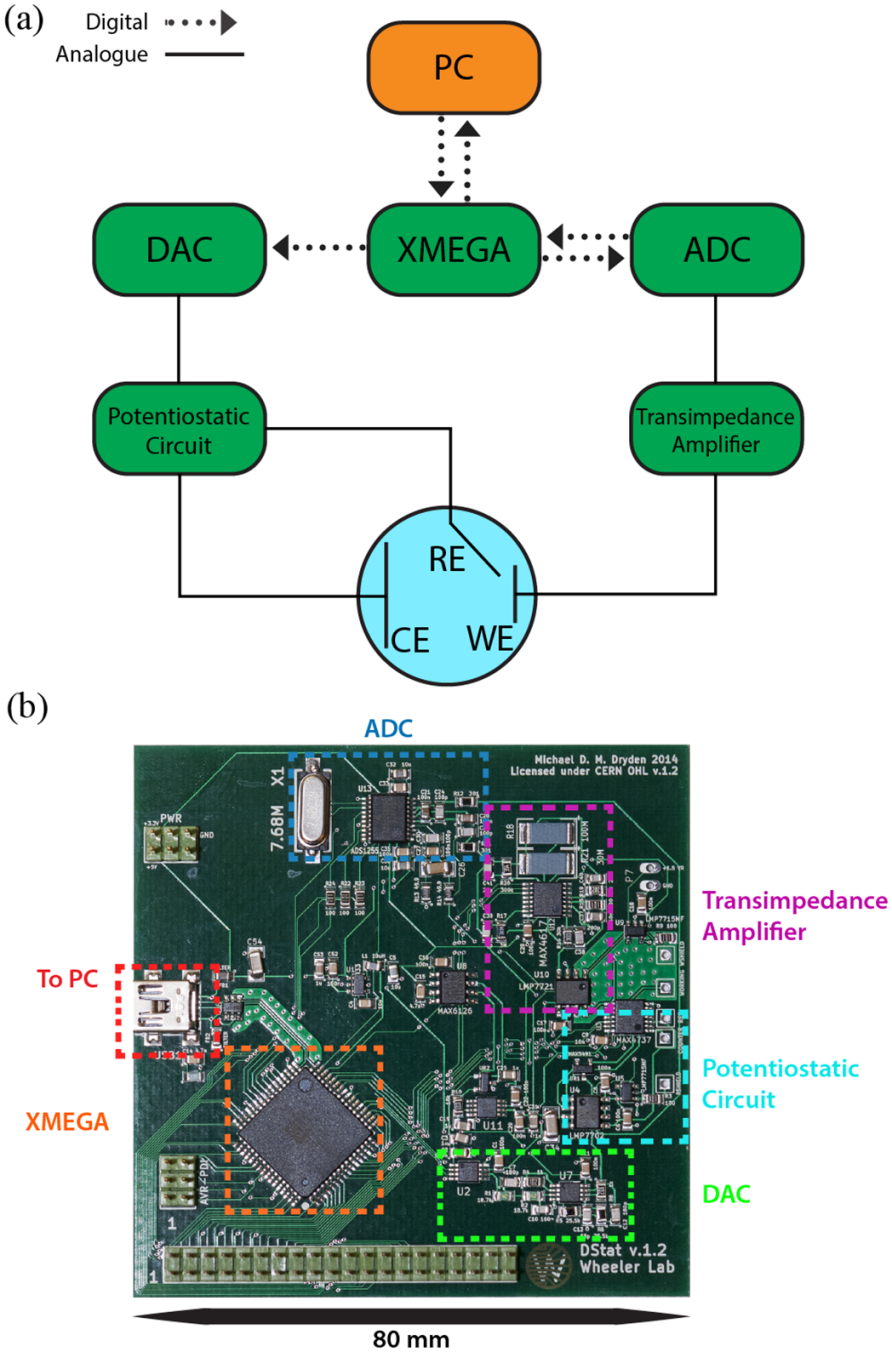
The DStat. (a) Schematic overview of key DStat components, including the computer (PC), the microcontroller (XMEGA), the analogue-to-digital converter (ADC), the digital-to-analogue converter (DAC), the potentiostatic circuit, and transimpedance amplifier. The DStat is interfaced to a three-electrode electrochemical cell, including a working electrode (WE), a counter electrode (CE), and a reference electrode (RE). Modules integral to DStat are coloured in green. Solid lines represent analogue connections. Dotted arrows represent digital connections. (b) Top-view picture of the DStat circuit board with labels corresponding to schematic components.

The capabilities of potentiostats vary greatly in terms of current capacity, potential range, resolution, etc., depending on their intended use. The hardware design goals for DStat were to allow amperometric and voltammetric measurements for conditions commonly found in research laboratories—modest currents at small electrodes, voltages typical of aqueous electrochemistry, and an emphasis on accurate low current measurements for sensing applications. Further, DStat was designed to be controlled and powered with a USB connection (providing both power and data), allowing portability for field use when combined with a battery-powered computer. Finally, considerations were made for ease of assembly and reduced cost (where possible) without compromising performance. DStat’s major components are shown in Fig 1 and an examination of each component follows. Additional details are found in S1 Supporting Information.

#### Potential control circuit

**Fig 2:**
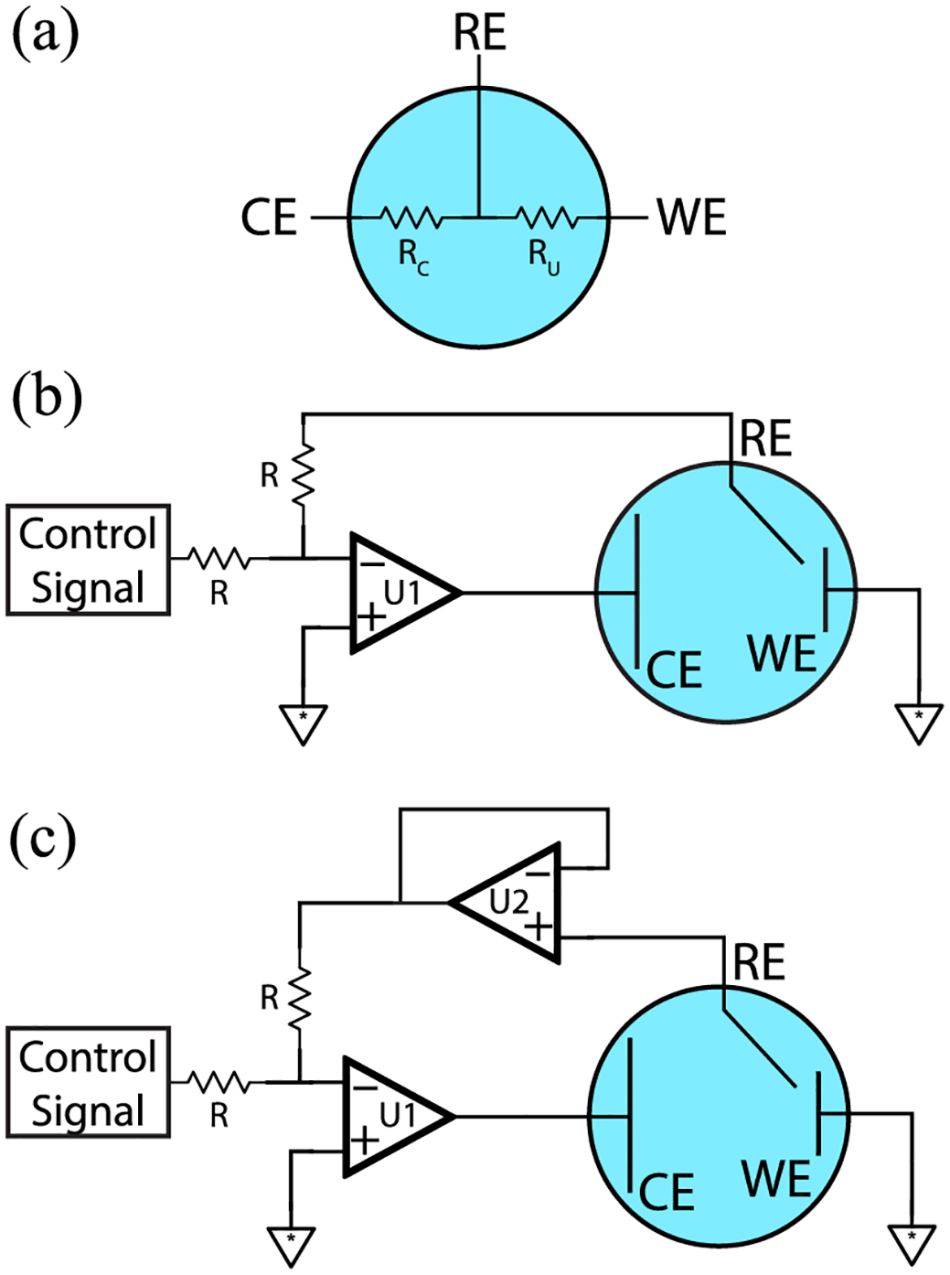
Electrochemical cells and potentiostatic circuits. RE: Reference Electrode, CE: Counter Electrode, WE: Working Electrode, R: Summing resistors, U1: Control amplifier, U2: Reference buffer amplifier, *R*_*c*_: Compensated cell resistance, *R*_*u*_: Uncompensated cell resistance. (a) Simplified three electrode cell model. (b) Basic potentiostatic circuit. (c) DStat potentiostatic circuit.

The most important function of a potentiostat is to control the interfacial potential at a working electrode (WE) in an electrochemical cell to allow for current to be measured at constant potential. This is typically achieved using a three-electrode cell in which a voltage is applied to a counter electrode (CE), which provides enough current to compensate for redox reactions occurring at the WE. This voltage is set by feedback from a reference electrode (RE). The use of a three-electrode cell provides two main advantages: (1) the RE is not susceptible to polarization error (where current flow results in a change in potential) and (2) the RE can be small enough to be placed very near the working electrode surface, minimizing potential error caused by solution resistance. As shown in Fig 2(a), a three-electrode cell can be modelled as two resistances in series, between CE and RE (*R*_*c*_) and between RE and WE (*R*_*u*_). A simple potentiostatic circuit relying on one op-amp (U1) is shown in Fig 2(b). As indicated, the RE is positioned in a negative feedback loop, and the op-amp output is applied to CE. This allows the op-amp to supply the current required to compensate for the IR drop across *R*_*c*_ (the compensated cell resistance). A working potential between RE-WE can be programmed by biasing the circuit with a control signal (described below) through the summing resistors (*R*). Thus, the only unwanted resistance in the circuit is *R*_*u*_ (the uncompensated cell resistance), which is kept as low as possible by positioning the RE close to the WE.

The circuit in Fig 2(b) is functional, but most modern potentiostats, including DStat, rely on a modified circuit, depicted in Fig 2(c). The main difference between Fig 2(b) and 2(c) is the addition of a second op-amp (U2), which serves as a unity gain buffer to limit any current that might otherwise flow through RE. This is particularly important given that the summing resistors typically have low resistance to minimize thermal noise (e.g., in DStat, *R* = 15 kΩ), meaning that significant current would pass through the reference electrode if not for U2. When potential control is not required U2 is also useful in (a) measuring a cell’s open circuit potential, and (b) buffering electrodes for potentiometric measurements, by bypassing U1 and measuring the output directly. The selection of op-amps U1 and U2 is critically important to the performance of the potentiostatic circuit and is detailed in S1 Supporting Information.

#### Control signal

Most potentiostats are designed such that the working electrode potential can be modulated as a function of time in a pattern dependent on the type of experiment, e.g., a linear change for linear sweep voltammetry. (Note that some lab-built potentiostats [7, 13] do not include this capacity, and thus require external control signals.) This control signal, which is applied to the negative feedback loop of the circuit, was historically produced using analogue circuits, a practice that is still in place in some lab-built potentiostats described in the recent literature. [9] But most modern potentiostats use a digital to analogue converter (DAC) to provide arbitrary control signals without needing electronic reconfiguration to change experiments.

A DAC can output only quantized voltage steps and therefore cannot represent every possible voltage within its output range. The difference between the intended output and the nearest DAC step is known as quantization error and varies with the intended output, reaching a maximum halfway between steps. The output range of a DAC is divided into 2^n^ steps where *n* is its resolution in bits. Quantization error was an important consideration in the design for DStat—for example, it would have been straightforward to use the 12-bit DAC that is included (by default) in the microcontroller (see Fig 1) used to control DStat. This practice was not adopted, however, as for the 0–3 V range targeted for DStat, a 12-bit DAC step in this configuration would be approximately 0.73 mV, resulting in a maximum quantization error of 0.37 mV. This magnitude of error can be problematic in applications that have small waveform amplitudes (e.g., see the square wave voltammetry data in the following sections). For this reason, we elected to use an external DAC with a resolution of 16 bits in DStat, resulting in a step size of 46 μV, an order of magnitude improvement over the 12-bit resolution of the microcontroller’s DAC. The quantization error for the 16-bit DAC-driven control signal in DStat is much lower than that of the DAC-controlled lab-built potentiostat circuits described previously (that rely on 4- [11], 8- [4], 10- [10], and 12-bit [3, 5, 8] DACs, respectively). The DAC’s sample rate and reconstruction filter are also important for producing accurate waveforms and are discussed in detail in S1 Supporting Information.

#### Current Measurement

**Fig 3:**
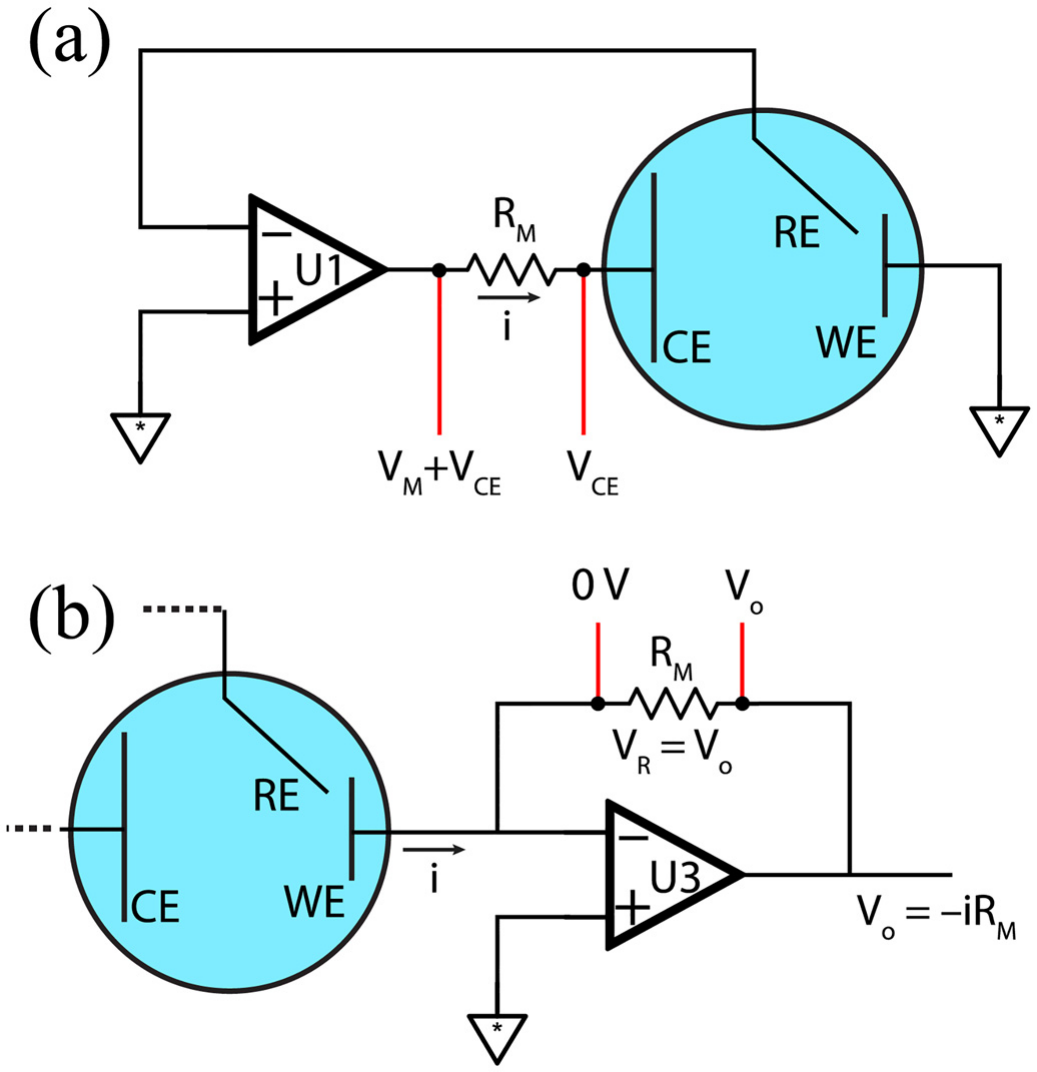
Cell current conversion to voltage for ADC. (a) Current measurement by shunt resistor. The measurement resistor *R*_*M*_ causes a voltage drop proportional to the cell current *i* by Ohm’s Law. The voltage drop is measured across the resistor but the counter electrode voltage *V*_*CE*_ (present on both sides of the resistor) complicates measurement. (b) Current measurement using a transimpedance amplifier. The measurement resistor *R*_*M*_ is placed in a negative feedback loop of an op amp (U3) whose inverting input is connected to the working electrode. U3’s non-inverting input is tied to ground, producing a virtual ground at the inverting input. When current *i* flows through the working electrode, it induces a voltage drop *V*_*R*_ across *R*_*M*_, which is balanced by U3 output *V*_*O*_ to maintain the virtual ground at its inverting input.

The potentiostatic circuit and control signal are sufficient to maintain a desired working electrode potential, but for almost all experiments involving a potentiostat, measurement of the current passing through the working electrode is required. Because the majority of analogue to digital converters (ADC) cannot measure a current directly, the current must first be converted to voltage to obtain a digital signal suitable for recording by a computer. The simplest technique to accomplish this (used in some of the home-made potentiostats described in the literature [14, 17]) is the current shunt, shown in Fig 3(a), where the voltage drop is measured across a measurement resistor *R*_*M*_ placed in series between the counter electrode and the control amplifier output. In practice, because ADCs cannot measure an infinitely large range of voltages, it is necessary to switch between different *R*_*M*_s to cover the several orders of magnitude of currents of interest in electrochemical experiments.

The current shunt approach has a number of disadvantages that make it undesirable for use in DStat: the output voltage is not referenced to ground (requiring the capability to make a differential measurement using an ADC that is tolerant of the counter-electrode voltage *V*_*CE*_ that is applied to both inputs); the measurement resistor forms part of the negative feedback loop, thus limiting the compliance voltage (i.e., the maximum voltage that op-amp U1 can produce to set the working electrode potential); and the measurement circuit must be designed to draw very little current to avoid influencing the measurement. To circumvent these limitations, DStat employs a transimpedance amplifier in series with the working electrode (Fig 3(b)). This type of circuit is commonly used for low current measurements, as the output voltage is provided by an op-amp and thus does not require high input impedance in the voltage measurement circuit. The conversion mechanism is similar to that of the current shunt, but *R*_*M*_ is in the negative feedback loop of a third op-amp (U3), to which the WE is connected via the inverting input. In this configuration, the measurement voltage is referenced to ground, allowing straightforward (non-differential) ADC measurement which does not affect the compliance of the potentiostatic amplifier (U1); and, if the transimpedance amplifier is set to have a maximum output that is the same as the maximum input of the ADC, no additional circuitry is required for protection.

Once the working electrode current has been converted to a voltage, it is passed to an ADC for measurement. Similar to DACs (described above), ADCs generate quantized measurements at discrete time intervals. The ADC’s voltage-resolution determines the size of the voltage increments between successive digital values. The ADC’s sample rate limits the highest frequency signal which may be measured; aliasing will occur if the analogue signal is not band-limited to below the Nyquist frequency. Resolution and sample rate also control the volume of data produced, an important consideration as data must be collected from the ADC, transmitted to a computer, and stored.

To aid in selection of an ADC for the DStat, the current measurement circuit was simulated (as described in the Experimental section) and the input-referred current noises calculated for each of 7 *R*_*M*_s from 100 Ω–100 MΩ. It was found that for any ADC resolution up to 16-bits (the maximum resolution reported for any lab-built potentiostat with integrated ADC reported previously [3–6, 8–11, 16, 17]) and all values of *R*_*M*_, the size of an ADC step is the limiting factor in overall resolution, rather than analogue noise. Therefore, to utilize the current to voltage conversion circuitry most efficiently, a 24-bit ADC (the highest commonly available resolution) was chosen for DStat. The particular ADC that was selected is of the sigma-delta type, which has several advantages for DStat: sampling occurs at a much higher frequency than the output rate so the requirements for the anti-aliasing filter are relaxed, and a digital filter is a part of the conversion process which can be adjusted to give either higher data rates or reduced noise measurements, depending on the needs of the experiment being performed. The digital filter can also be used to reduce environmental noise pickup (especially 50/60 Hz mains noise) by matching the output sample rate to the noise frequency as the filter has zeros in its frequency response at multiples of the output sample rate.

#### Microcontroller

The analogue components of a potentiostat may place absolute limits on performance, but to make full use of the instrument’s capabilities, it must be possible to update the DAC at the necessary rate, acquire ADC data as it is collected, and send it to a computer for recording. The requirements of these tasks vary with the type of experiment being performed, but it is often necessary to run all three tasks nearly simultaneously; e.g. for a cyclic voltammetry measurement, the voltage must be swept continuously by the DAC, ADC measurements must be collected as they are produced, and data must be sent to a computer for storage and processing as it is impractical to store the data from many cycles on the instrument. One method for performing these functions is to use a “virtual instrument” in which a computer commands and receives data from a so-called “data acquisition” device containing integrated DACs, ADSs, and switches (e.g., the CompactDAQ system—National Instruments, Austin, TX). These kinds of devices are useful for prototyping and rapid instrument-assembly, and have been used in some lab-built systems described in the literature. [15–17] But systems relying on these kinds of devices are limited in that the electronics must be connected externally (limiting the robustness and portability of the assembled system), computer requirements are more stringent because of proprietary software and the necessity for direct control over peripherals, and flexibility is lost in (a) the selection of components tailored to the intended use of the instrument, and (b) the difficulty of separating the computer software from the instrument hardware (which can hinder integration with other instruments). Another option for controlling the potentiostat hardware is an integrated microcontroller. [19] A microcontroller is essentially a low-power computer processor (CPU) coupled with memory and other peripherals such as data converters and communications modules, intended for embedded use—performing specific tasks as part of a larger device, rather than being suited for general use. To accomplish these tasks, DStat makes use of an ATxmega256A3U microcontroller (Atmel Corporation, San Jose CA, USA), chosen to balance performance with cost.

In DStat, the microcontroller manages the digital components of the instrument—the DAC, ADC, and switches for connecting the electrochemical cell and setting the *R*_*M*_. Additionally, the microcontroller features integrated USB support, obviating the need for a separate module to communicate with a computer. Because DStat is not intended to operate without a computer connection, any data manipulation such as conversion of ADC and DAC units to amperes and volts or digital processing are done on the much more powerful computer. Details of the software implementation of electrochemical experiments and strategies to limit processor loading are discussed in S1 Supporting Information.

Some previously reported lab-built potentiostats also made use of microcontrollers. [3–6, 8–10] Notably, the Cheapstat [8] uses a different model of Atmel microcontroller; DStat’s model has more memory (and allows for more complex firmware), and in addition allows for convenient, internal USB connection (not requiring an external chip).

### Reagents and materials

Analytical purity reagents were purchased from Sigma-Aldrich Canada Co. (Oakville, CA). Fresh solutions were prepared as needed using 5.6 μS/m deionized water. Measurements of potassium hexacyanoferrate (III) were performed on DRP-223AT disposable screen printed electrode sets with gold working and counter electrodes and a silver pseudoreference electrode purchased from DropSens (Llanera, Spain). 4-aminophenol solutions were measured using microfabricated electrode devices on silicon substrates. These devices feature an array of 20 gold nanostructured microelectrode working electrodes formed as described previously [20] and two rectangular gold electrodes that serve as counter and pseudoreference electrodes for the array of working electrodes. The working electrodes were measured separately, rather than as an ensemble, to highlight instrument performance at low current levels.

### Electrochemical Control Systems

DStat was built as described per the parts lists and assembly instructions in S1 File, operated by running the custom software on a laptop via USB connector. The most recent version can be retrieved from http://microfluidics.utoronto.ca/dstat. A CheapStat was built and operated as described previously [8], and an EmStat 1 and an Accumet AR50 were used as received from PalmSens BV (Utrecht, NL) and Thermo Fisher Scientific (Waltham, MA, USA), respectively.

### TINA-TI simulations

TINA-TI version 9 (Texas Instruments and DesignSoft Inc.) was used to simulate DStat’s current measurement circuit. The DC absolute current error was set to 50 fA and the DC absolute voltage error was set to 1 nV, while other analysis parameters were left at default levels. The software’s noise analysis function was used to calculate the total noise between 16 mHz and 60 Hz (corresponding to the open circuit noise measurements described below), for each value of *R*_*M*_.

### Open circuit noise measurements

To quantify the noise of the current measurement circuit, the working electrode input was left open and a 60 s current measurement was acquired for each *R*_*M*_ at a sample rate of 60 Hz (to reflect typical measurement conditions and reduce AC mains noise, as described above). The noise value was taken as the standard deviation of the data. The measurements were performed in a typical laboratory setting without special precautions to reduce environmental noise (e.g., enclosure in a Faraday cage).

### Potassium hexacyanoferrate (III) measurements

A 10 mM solution of potassium hexacyanoferrate (III) in pH 7 McIlvaine buffer [21] was prepared and used for measurements on the screen printed electrodes. The electrode substrate was clamped horizontally and a drop of measurement solution (∼ 100 μL) was pipetted onto the electrode cell. A new electrode cell was used for each potentiostat as repeated measurements were observed to alter electrode response. A fresh droplet of solution was used for each measurement after washing the electrode cell with deionized water three times, removing the previous droplet with a paper wipe.

Cyclic voltammograms were recorded from -250 to 520 mV at a scan rate of 100 mV/s. DStat’s sample rate was set to 60 Hz and the gain fixed to 30 kV/A. The EmStat’s sample rate was set to 1 mV/sample (100 Hz) and was allowed to switch range settings between 1 and 100 μA full scale. The CheapStat’s gain was set to 27.5 kV/A and (because of its limited storage capacity) samples were only acquired every 6 mV. Five sequential scans were acquired by each potentiostat and the ultimate scans used for the comparison.

Square wave voltammograms (SWVs) were recorded from -150 to 500 mV with a step height of 2 mV, an amplitude of 25 mV, and a frequency of 70 Hz. DStat’s sample rate was set to 500 Hz and neither the EmStat nor the Cheapstat have provisions for controlling sampling time during SWV experiments. All three potentiostats’ gain settings were the same as for the cyclic voltammetry experiments (above). Only a single scan was acquired for each electrode cell as repeated SWV of the measurement solution was observed to affect the electrode response.

### 4-aminophenol measurements

Four solutions of 4-aminophenol (100, 50, 25, and 10 μM) were prepared in 50 mM pH 9 Tris-HCl buffer for measurement with the microfabricated electrode devices. The devices were clamped horizontally and 30 μL of a measurement solution was pipetted to cover the electrode surfaces. Four working electrodes were used for each potentiostat with a single droplet of measurement solution. The working electrodes were measured in sequence before replacement of the droplet, with rinsing as described for the potassium hexacyanoferrate(III) measurements.

Differential pulse voltammograms were acquired from -300 to 250 mV with a step size of 5 mV, a pulse height of 50 mV, a pulse period of 100 ms, and a pulse width of 50 ms. DStat performs differential pulse voltammetry similarly to SWV and the sample rate was set to 500 Hz as above, with a gain setting of 300 kV/A. The EmStat was allowed to switch gains between 1 nA and 10 μA full scale. The CheapStat was not able to produce a recognizable peak at any available setting and was thus excluded from the experiment. Data was normalized by subtracting the current of each measurement at -150 mV from each sample to correct for changes in background current between measurements.

## Results and Discussion

### Noise measurements

**Fig 4:**
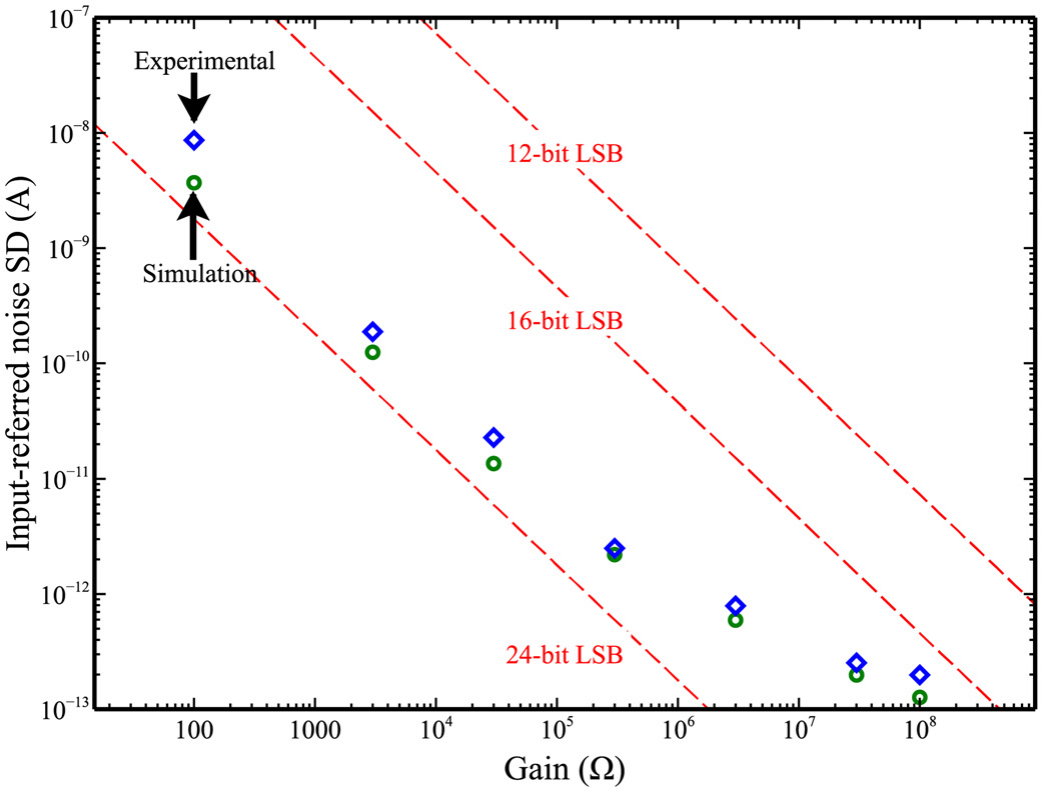
Open circuit DStat input-referred measurement noise standard deviations (SD) as a function of gain (set by the different DStat *R*_*M*_s) at a sample rate of 60 Hz measured over 60 s. Blue diamonds are experimental measurements and green circles are TINA-TI simulation results. The equivalent input currents of a least significant bit (LSB) for three different ADC resolutions are shown as dashed red lines.

One of the most important performance specifications of a potentiostat, particularly for one that is intended for use in analytical measurements, is the ability to precisely measure small currents. With the advent of nano- and micro-scale electrodes, this is particularly important as the current response of a single electrode may be in the pA—nA range. To establish a performance level for the current measurement system, DStat’s open-circuit noise level (without a connected electrochemical cell) was measured. The measured standard deviations (blue diamonds) are plotted in Fig 4 as the equivalent current at the working electrode input (representing the analogue noise in the system) as a function of gain (set by the values of *R*_*M*_). For comparison, the currents associated with a single voltage step in a 12-, 16-, and 24-bit ADC are also plotted as dashed red lines. As shown, higher resolution ADCs have smaller steps (and thus can measure lower currents), and the size of each step decreases with increasing gain (as it takes a smaller current at the working electrode to produce a given output voltage at the transimpedance amplifier). Thus, for a specified gain and ADC resolution, the smallest current that can be measured is limited by either the size of the ADC’s steps or the analogue noise of the measurement circuit. Lower resolution ADCs, such as the 16- and 12-bit circuits represented in the figure, limit the precision of the current measurement at all gain settings and thus cannot achieve the resolution of which the analogue circuitry is capable. That is, the dashed lines for the 12- and 16-bit ADCs are far above the level of the analogue noise for all levels of gain. The utility of the DStat’s 24-bit ADC is clearly demonstrated in the figure as this circuit enables sufficient resolution at all gain settings to avoid truncating data above the analogue noise of the measurement circuit—that is, the dashed line for the 24-bit ADC is below the level of the analogue noise at all values of *R*_*M*_.

As shown in Fig 4, DStat’s measured analogue noise (blue diamonds) decreases with increasing gain before levelling off at approximately 200 fA (approaching the largest value of *R*_*M*_), placing the instrumental limit of detection at approximately 600 fA. (The experimentally observed noise is closely mirrored by the TINA-TI simulated values shown in green circles; see S2 Supporting Information for additional discussion of the trend in noise.) A lower current measurement limit in the hundreds of femtoamperes is sufficient for the currents of most experiments and DStat’s noise performance is unlikely to be limiting except in extremely low current measurements in well-shielded measurement cells. Note that this value (600 fA) is approximately five orders of magnitude lower than the only one of the lab-built potentiostats described previously that reported a comparable value (i.e., Cumyn et al. [14] reported a noise limit of 10 nA).

### Electrochemical performance

**Fig 5:**
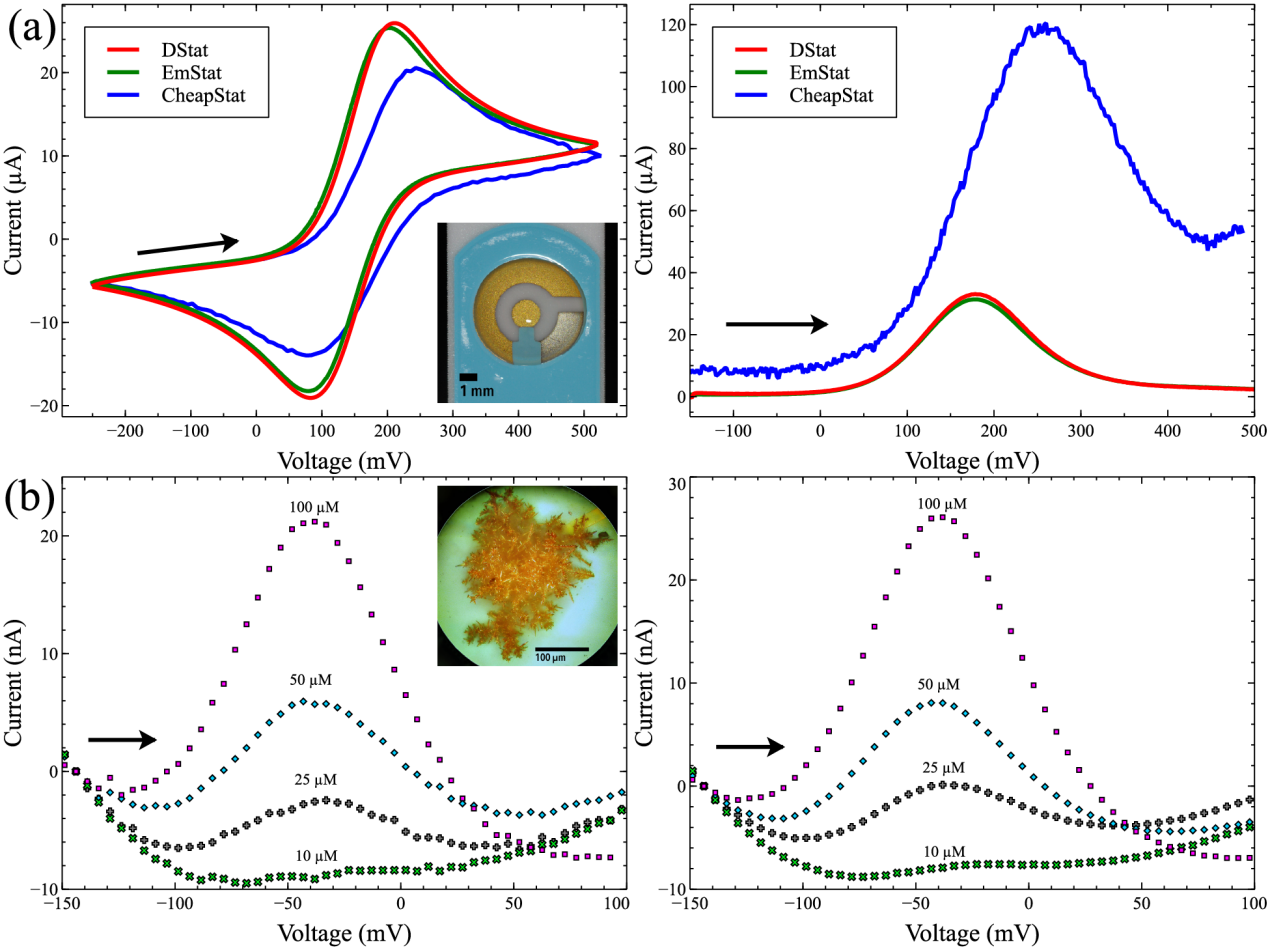
Comparison of voltammetric measurements between instruments. Arrows indicate scan directions. (a) Cyclic voltammetry (left) and square wave voltammetry (right) of 10 mM potassium hexacyanoferrate (III) collected using (commercial) screen printed electrodes. DStat (red traces), EmStat (green traces), and CheapStat (blue traces). Inset: picture of a screen printed electrode. (b) Differential Pulse Voltammetry of 4-aminophenol (10–100 μM) with DStat (left) and EmStat (right) collected using nanostructured microelectrodes. Curves were shifted vertically to align the current at -150 mV to 0 A to correct for changes in background current. Inset: picture of a nanostructured microelectrode.

DStat’s voltammetric performance was compared to that of two peer-systems: an EmStat purchased from PalmSens BV (Utrecht, NL), and a CheapStat constructed following the instructions provided by Rowe et al. [8] Each of these systems is inexpensive (DStat ∼ $120 CAD/materials, EmStat ∼ 1000 EUR/unit, CheapStat ∼ $60 CAD/materials), portable (DStat 92×84×31 mm plus laptop, EmStat 61×43×16 mm plus laptop, CheapStat 140×66×28 mm), and is designed to enable multimodal electroanalytical analysis in the lab or in the field. DStat and CheapStat are open source; EmStat operates as a black box. Fig 5(a) shows representative cyclic voltammograms and square wave voltammograms of 10 mM potassium hexacyanoferrate(III), an analyte exhibiting a chemically reversible reduction under most conditions. While all three potentiostats produced the classic double peak-shaped cyclic voltammograms (left) and the single well-defined peak on the square wave voltammograms (right) expected of a reversible redox couple, some differences were observed between instruments. The DStat and EmStat displayed almost identical voltammograms, characterized by smooth responses which were free of noise; the slight differences between them likely caused by variations in individual electrodes. In contrast, the voltammograms of the CheapStat are significantly different: the CheapStat’s cyclic voltammogram has unusually wide peak separation and reduced peak heights, and its square wave voltammogram peak is asymmetric, with significant background current. Further, both of the CheapStat’s voltammograms are visibly noisy.

The distortions in the CheapStat peak shapes may be caused by the limited compliance voltage of the control amplifier (∼ ±1 V) or the bandwidth of its transimpedance amplifier—at the square wave frequency selected, 70 Hz, TINA-TI simulation indicates that the CheapStat’s amplifier’s frequency response rolls off above 90 Hz, severely distorting signals with higher frequency components. The observed noise in the CheapStat’s voltammograms may be caused by the use of the microcontroller’s on-board 12-bit DAC as a reference for the ADC: the DAC is itself referenced to the system’s 3.3 V power line which may be subject to more noise than DStat’s precision reference voltage source. In addition, the increased noise in the CheapStat’s voltammograms could be related to the aliasing effects observed in this system (See S1 Supporting Information). Regardless, it is apparent in the case of the relatively large currents of the screen printed electrodes that DStat exceeds the capabilities of the expense-oriented CheapStat and is indistinguishable from the commercial EmStat instrument.

To examine low-current applications, differential pulse voltammetry measurements of 4-aminophenol were performed using a custom sensor bearing integrated gold nanostructured microelectrodes (NMEs). [20] As shown in the inset to Fig 5(b), the large surface areas of these specialized features allows for high-density attachment of antibodies and other probe molecules, which has attracted significant attention in the biosensor community. [22–24] But the improvement in sensitivity for NMEs comes at a cost—the small overall dimensions of NMEs are associated with small redox currents, requiring more sensitive instrumentation than what is needed for (more standard) experiments using screen printed electrodes. NMEs were used here as unmodified WEs to evaluate the conversion of 4-aminophenol to 4-quinoneimine at four concentrations from 10 to 100 μM. As shown in Fig 5(b), DStat and EmStat produced very similar voltammograms with peaks located at identical voltages within the resolution of the experiment. Note that the DStat traces appear to have slightly higher levels of noise; however, it seems that the “black box” software of the EmStat performs an uninterruptible data-smoothing routine after the experiment is complete, making comparison of unfiltered signals impossible. Access to unfiltered data is useful for scientists who wish to apply (and report) their own processing routines prior to sharing or publication. Nevertheless, both the EmStat and DStat are clearly compatible with low-current measurements using NMEs, while the CheapStat did not produce recognizable oxidation peaks at these current levels.

Neither of the analytes probed in the voltammetry experiments described here (Fig 5) were measured in conditions with high cell capacitance or RE resistance, but DStat’s performance under such conditions (which favour instability of the potentiostatic circuit) is discussed in S3 Supporting Information.

Finally, DStat was designed to be compatible with current-measuring applications such as voltammetry (as per Fig 5), but also with voltage-measurement applications such as potentiometry. While most commercial potentiostat systems (including EmStat) are compatible with potentiometry, only one of the lab-built systems described previously [3] has this capability. To evaluate DStat’s capabilities for potentiometry, measurements of pH calibration standards were collected and compared with those generated using an Accument AR50 benchtop pH meter. As shown in S4 Supporting Information, the two instruments’ performances are virtually indistinguishable—both demonstrated the expected Nernstian response with root mean square errors from a linear least squares fit of 1.9 mV and 2.3 mV for the AR50 and DStat, respectively, which lies within the normal variation of a glass pH electrode. Further, because DStat samples at up to 30 kHz (compared to the 1 Hz sampling of the AR50), we propose that DStat may also be useful in applications that can take advantage of fine temporal resolution such as flow potentiometry [25] and carbon nanotube based sensors [26].

### Extensibility

A great advantage that open-source instruments often have over commercial instruments is complete access to both the electronic hardware and the software used to operate it, giving exceptional flexibility in extending the instruments to new uses. [18] While the EmStat offers a software development kit to allow access to built-in features from third party software, the hardware design and firmware (software that runs within the instrument) of the instrument are not disclosed. Conversely, for DStat, the full hardware schematics and source code for the computer software and instrument firmware are freely available (http://microfluidics.utoronto.ca/dstat) for modification or integration into other projects. This means, for example, that it is trivial to adapt the existing DStat program for square wave voltammetry into a custom routine for cyclic square wave voltammetry, with the addition of less than 5 lines of code to the firmware. Similarly, programs for small amplitude and large amplitude pulse voltammetry, specialized techniques that are used for voltammetric electronic tongues [27], can be implemented by simple modifications to the existing firmware.

Perhaps most importantly, DStat software can be connected to third-party software, permitting the use of the standard DStat user interface, triggered by signals from another program. This functionality uses the open-source ZeroMQ distributed messaging system (http://zeromq.org) to provide simple connectivity between the DStat software and other programs running on the same computer or across a network. The hardware can also be controlled directly by means of a simple emulated serial interface over USB.

**Fig 6:**
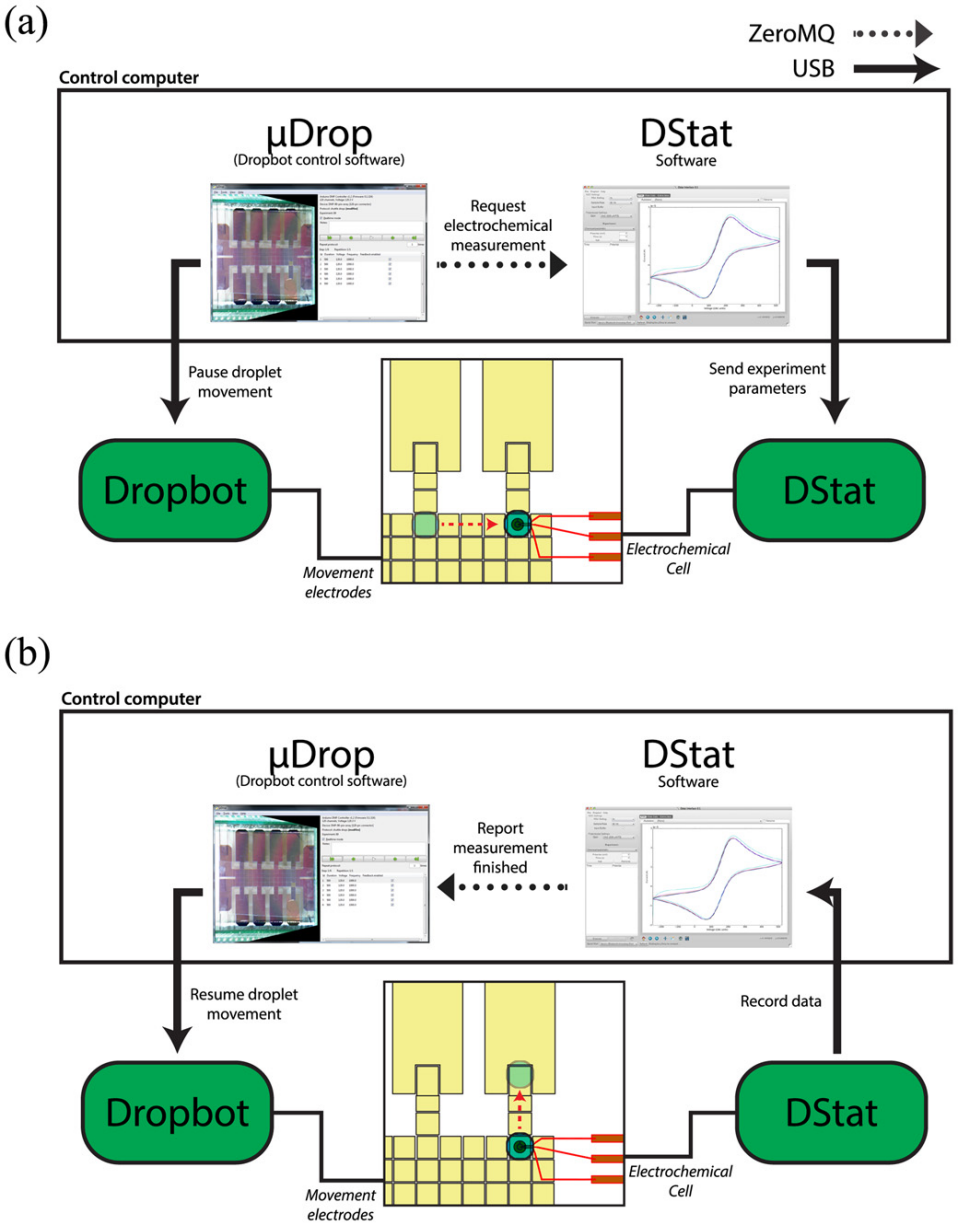
DStat/Dropbot integration. Solid arrows represent communication between the control computer and instruments over USB and dotted arrows represent communication between programs within the control computer over ZeroMQ. (a) When Dropbot’s control software μDrop reaches a programmed electrochemical measurement step, it pauses droplet actuation (in the figure, represented by a droplet parked at a circular electrochemical cell similar to the one described by Dryden et al. [31]) and requests an electrochemical measurement from the DStat software. The DStat software processes the request by initiating an experiment on the DStat hardware. (b) As the DStat hardware performs the experiment, the DStat software records the data. When the experiment is complete, the DStat software reports to μDrop that the measurement is complete and μDrop resumes its programmed droplet movement. A movie depicting the full process can be found in S1 Video.

To demonstrate the flexibility of DStat, a simple plug-in for the open-source Dropbot digital microfluidics control system [28] was created, providing automated electrochemical analysis including programmable microscale fluid manipulation. Fig 6 illustrates the operation of the plugin: μDrop, the control software for Dropbot operates by iterating through a list of pre-programmed steps of droplet manipulations. The new plug-in (which can be found at http://microfluidics.utoronto.ca/dstat) allows the creation of special steps in which droplet manipulation is paused and a request for an electrochemical measurement is sent to the DStat software using ZeroMQ, which then initiates an experiment on DStat (Fig 6(a)). When the DStat experiment is complete and the DStat software has recorded the data, a signal reporting a finished measurement is sent back to μDrop via ZeroMQ, allowing μDrop to resume droplet manipulation (Fig 6(b)). A video demonstration of combined droplet manipulation and electrochemical measurement can be found in S1 Video with experimental details in S5 Supporting Information. Thus, the extensibility of DStat makes it an attractive tool for the burgeoning area of digital microfluidic electrochemistry [29–33].

The DStat joins the growing ranks of inexpensive, freely-modifiable open-source analytical instruments that both complement and compete with commercial instruments. We propose that DStat will prove compatible with a wide range of customized lab-built systems, such as the PublicLab spectrometer (http://publiclab.org/wiki/spectrometer) and miniature SPR systems [34], as well as commercial devices including microfluidic flow controllers, autosamplers, and chromatography instruments. DStat is also applicable beyond electrochemical cells, for use with photodiodes, photomultiplier tubes, Faraday cups, and other devices requiring measurement of minuscule currents.

## Conclusions

The DStat joins the growing ranks of inexpensive, freely-modifiable open-source analytical instruments that both complement and compete with commercial instruments. We demonstrated its capabilities for low-current voltammetry in laboratory settings and found its performance to be comparable to that of a compact commercial potentiostat as well as being superior to that of a previously reported open-source instrument. DStat was also demonstrated to be useful for potentiometry (mostly unique among lab-built potentiostats) with performance comparable to that of a commercial pH meter. Finally, DStat provides robust integration with other instruments by allowing either direct communication with the hardware or using the DStat software as intermediary, which was demonstrated by connecting the DStat software with the Dropbot digital microfluidics platform. With this contribution to the toolkit of open-source analytical instruments, we hope to foster free modification and allow researchers to adapt their tools to suit experiments rather than alter experiments to suit tools.

## Supporting Information

S1 Supporting Information Hardware considerations. Additional details concerning operational amplifier selection, DAC output quality, power supply, and obtaining optimal microcontroller performance.
(PDF)

S2 Supporting Information Analysis of noise measurements. Discussion of noise properties of current measurement circuit.
(PDF)

S3 Supporting Information Potentiostatic circuit stability. Examination of the stability of the potentiostatic circuit under capacitive loads.
(PDF)

S4 Supporting Information Potentiometry. Demonstration of DStat’s potentiometry capabilities by pH measurements and comparison with a commercial pH meter.
(PDF)

S5 Supporting Information Integration of DStat with Dropbot. Experimental details of DMF device fabrication and device operation for integration with DStat.
(PDF)

S1 Video Demonstration of DStat-DropBot interaction. A 1.5 μL droplet of 10 mM potassium hexacyanoferrate(II) is dispensed from a reservoir and translated to the electrochemical cell, followed by a cyclic voltammetry measurement by DStat. Upon completion of the measurement, the droplet is pulled away from the cell. (The recording of the DStat measurement is accelerated 4x for brevity.)
(MP4)

S1 File Hardware and software design files. Electronics manufacturing files, software and firmware source code, and documentation for DStat construction and operation. The most recent version can be retrieved from http://microfluidics.utoronto.ca/dstat.
(ZIP)
