# Supplementary material for: DStat: A Versatile, Open-Source Potentiostat for Electroanalysis and Integration
Source: PLoS One. 2015 Oct 28;10(10):e0140349. doi: 10.1371/journal.pone.0140349 (PMC4624907; doi:10.1371/journal.pone.0140349)
Supplement: S1 Supporting Information — Additional details concerning operational amplifier selection, DAC output quality, power supply, and obtaining optimal microcontroller performance. (PDF) [file pone.0140349.s001.pdf]

S1: Hardware considerations

Operational amplifier selection

DStat relies on three op-amps: the control and buffer amplifiers in the potentiostat (U1 and U2 in Fig. 2(c) in the main text) and the transimpedance amplifier in the current measurement circuit (U3 in Fig. 3(b) in the main text). In DStat, these amplifiers are the LMP7702 (Texas Instruments, Dallas TX, USA) for control and buffer and LMP7721 (Texas Instruments) for transimpedance, respectively. These models were carefully selected on the basis of a number of criteria. To keep polarization error small, the buffer was chosen to have a minuscule input bias current (~200 fA; a function of its CMOS input stage), allowing it to maintain a negligible reference electrode current. To avoid problems with potential inaccuracies, control and buffer amplifiers with large open loop gains and small voltage noise densities and input voltage offsets were selected, as a closed loop gain error exists that is the reciprocal of the open loop gain (for an inverting amplifier with gain of -1), and potential accuracy is also affected by the amplifiers' voltage noise densities and input voltage offsets. Additionally, the control and buffer amplifiers' bandwidths should be sufficiently high to accommodate the potential roles of the potentiostat—a bandwidth of a few hundred kilohertz may be ample for typical experiments at macroelectrodes, but several megahertz may be required for fast scanning experiments or electrochemical impedance spectroscopy. In DStat, the LMP7702 fulfils the requirements for both amplifiers while reducing design complexity by containing two amplifiers in the same physical package. Further, the LMP7702 has an open loop gain of approximately 10^6, an input bias voltage of ± 32 µV, and an input referred voltage noise density of 9 $\mathrm{nV}/\sqrt{\mathrm{Hz}}$ (at 1 kHz), allowing potential accuracy to tens of microvolts, and with a unity gain bandwidth of 2.5 MHz, potential control can be maintained at high enough frequencies for DStat's intended uses.

In the transimpedance measurement circuit, the input bias current of the op-amp is summed with the current at the working electrode input and can cause a large offset error, especially at high gain. For example, a simple op-amp such as the Texas Instruments LM741 can have an input bias current as high as 500 nA, a non-trivial error for low-current voltammetry; if this amplifier were used, the measurement circuit would cause output saturation at gains over 3 MΩ (for a ±1.5 V output range) without an applied current, making measurements impossible. Further, input bias current can vary significantly between op-amps of the same model, which complicates calibration, and electronic compensation requires manual tuning, increases noise, and may not be able to fully remove input bias current. To overcome these challenges, we chose the internally-compensated LMP7721, which has an extremely small input bias current of 3 fA (~1 electron every 53 µs), effectively eliminating bias current error.

DAC sample rate and reconstruction filtering

As described in the main text, DACs are an integral part of most modern potentiostat control circuits. The MAX5443 (Maxim Integrated, San Jose CA, USA) DAC was selected for DStat to allow implementation of appropriate filters and to minimize aliasing. DACs are discrete time devices—only able to change value at finite time intervals. The rate at which the value can be updated is crucial for several reasons. Most significantly, the Nyquist-Shannon sampling theorem reveals that in order to reconstruct a continuous signal of a given frequency from discrete samples, samples must be output at at least double the frequency of the signal. Additionally, for correct reconstruction, the output must be band-limited to remove frequencies above the Nyquist frequency (half the sample rate) or else artifacts will be observed as mirrored and normal spectral duplicates of the output spectrum (known as images), at multiples of the sampling frequency, in the frequency domain. That is, if the output is not band-limited, images will be found at $nF_s \pm f$, where n is a positive integer, F_s is the sample frequency, and f is the frequency of a signal in the intended output spectrum. The reconstruction filter's function is mainly to remove these images and should ideally have the response of a rectangular function in the frequency domain, such that all frequencies below the Nyquist frequency are passed completely and all higher frequencies are completely blocked. Unfortunately, it is not possible to have a filter with an infinitely high stopband rolloff; therefore, the sample frequency must be sufficiently high to allow a gap between

the highest frequency component of the output spectrum and the Nyquist frequency. In addition, most DACs operate with a zero-order hold, where a given output voltage is maintained until the next value is set, resulting in a staircase-like output that results in an attenuation of high frequencies corresponding to $|\frac{\sin(2\pi F_s)}{2\pi F_s}|$. This can be corrected by analogue or digital filtering, but the simplest way to ensure accuracy at high frequencies is to use a sample frequency much higher than the highest desired output, since the attenuation is relatively small at low frequencies. Using a high sample frequency also relaxes the requirements for the reconstruction filter since there is a large gap between the highest output frequency and the Nyquist frequency.

The effects of aliasing are shown in Fig. A(a) where the sine wave outputs from DStat and CheapStat at 70 Hz with an amplitude of 50 mV are compared. CheapStat has a relatively low sample frequency of approximately 2 kHz and doesn't employ a reconstruction filter, resulting in obvious jumps between samples. These images manifest as peaks at multiples of the sampling frequency in the frequency domain (Fig. A(b), left). This can be problematic for voltammetry since the sudden voltage changes will result in transient spikes in the non-faradaic current at the working electrode, distorting the measurement, depending on the cell capacitance, and desired measurement speed. These aliases are a natural part of a DAC's output and are also visible in DStat's (raw) output recorded without applying the reconstruction filter (Fig. A(b), right—green curve). To compensate for the images, DStat's DAC employs an active 4th order low pass filter with a cutoff frequency of 35 kHz, constructed around a MAX4477 (Maxim Integrated) dual op-amp. This leaves ample usable bandwidth with the DAC's maximum attainable sample rate of ~100 kHz while removing the majority of the images visible in the frequency domain (Fig. A(b), right—red curve) resulting in the smooth curve in Fig. A(a). The filter also prevents voltage glitches that can occur when switching a DAC's output from being applied to the cell.

Power supply constraints

DStat was designed to be a self-contained instrument that is capable of operation with only connectors for electrodes and a computer and power connection over USB. Thus, DStat can be used anywhere a computer can be operated, even in the field using a laptop on battery power. DStat's analogue components were chosen to be compatible with the 5 V supplied by the USB connection, but the digital components of the instrument require 3.3 V. In DStat, this is supplied by a low-dropout regulator (TPS79933, Texas Instruments). A high precision voltage reference (MAX6126, Maxim Integrated) provides an accurate 3 V source with which to reference analogue components. The reference is also used to power a 1.5 V line, formed using a precision voltage divider (MAX5491, Maxim Integrated) and a low noise op-amp buffer (OPA350, Texas Instruments) which serves as a signal ground for the analogue circuitry. This restricts the full potential range of the potentiostat to ±1.5 V (relative to the signal ground), sufficient for most aqueous voltammetry applications.

Reducing microcontroller loading

As described in the main text, DStat relies on an ATxmega256A3U microcontroller (Atmel Corporation, San Jose CA, USA) to manage the DAC and ADC, and to switch the measurement resistors. Criteria evaluated in selecting this component included the interrupt controller and event and timing control, features that allow tasks to be performed concurrently without overloading the CPU. The interrupt controller is a common feature found on microcontrollers that allows the normal program flow to be interrupted by certain signals, either from external sources (from other chips on the board, over USB) or from internal peripherals. When an interrupt is triggered, the main program is suspended, a section of code known as an interrupt handler (specific to each interrupt) is executed, and then the main program resumes from where it was suspended. In DStat, an interrupt can be triggered when the external ADC signals that data is ready, sending the data to the computer, which allows the CPU to handle other tasks rather than constantly polling the ADC to check if data is ready. An interrupt is also used to set new DAC values, triggering when a timer overflows (see below) indicating that a new value should be set. The interrupt controller of the xmega series microcontrollers is particularly flexible in that it permits different priorities to be set for each interrupt. In DStat, this is used to allow data to be read from ADC before a subsequent measurement occurs, without being interrupted by DAC writes, which are not timing critical.

A second common microcontroller feature is the timer/counter (TC). TCs are peripherals that store a number that can be incremented or decremented without use of the CPU and will overflow when it reaches its minimum or maximum value, continuing to count from the other value. A TC's current value can be read or set explicitly and can be used as either a timer or a counter, depending on its source. When used as a timer, the TC increments automatically at a fraction of the CPU frequency, and by setting the timer's maximum value, the time between overflows can be controlled. When used as a counter, the TC is incremented manually when called by the CPU or can be incremented by the overflow of a timer (a special feature of the xmega

series). DStat uses TCs to perform many functions without constantly loading the CPU. For example, (a) timers are used to keep track of when the value of the DAC should be changed and for providing elapsed time values for measurements, and (b) counters are used to store the current DAC value, incrementing or decrementing automatically when the DAC timer overflows, to produce a linear voltage output for linear scan voltammetry and cyclic voltammetry.

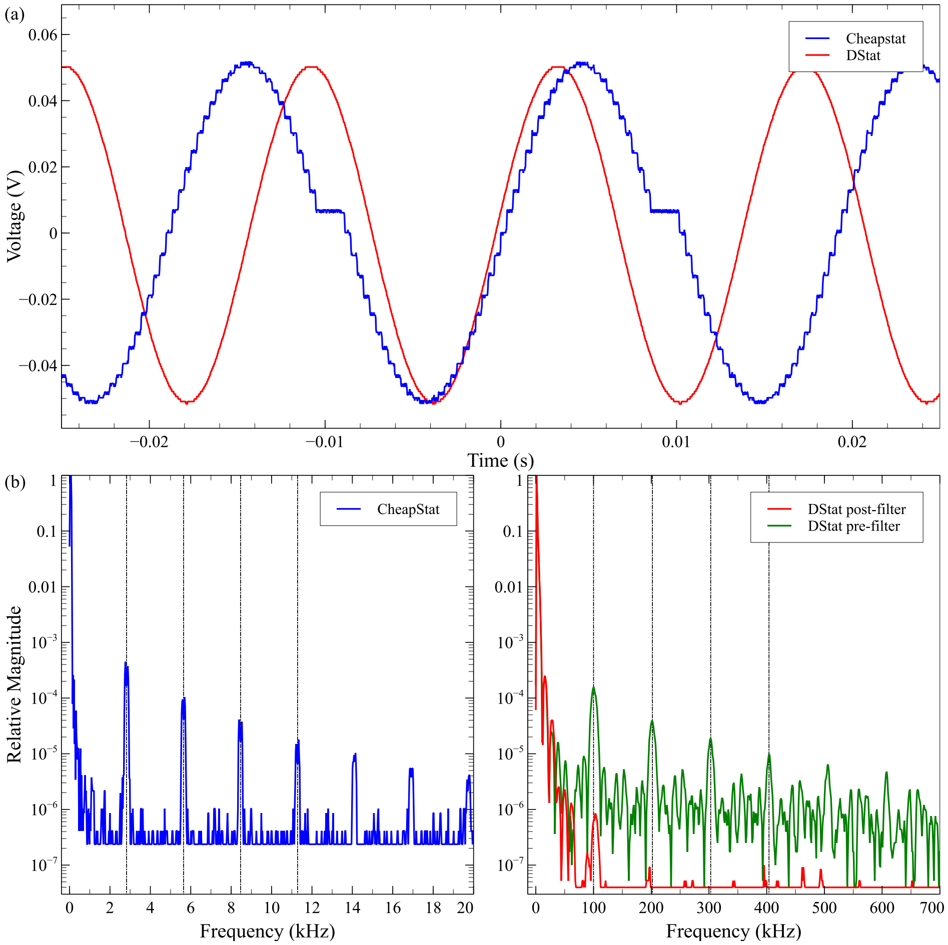

Figure A. Comparison of DAC-programmed control signals in DStat and CheapStat for a sine wave output at 70 Hz with an amplitude of 50 mV. (a) Time domain output of DStat (red) and CheapStat (blue). The CheapStat output shows harmonic distortion from a small delay between periods as well as visible aliasing. (b) Frequency domain output of the CheapStat (left) and DStat (right). Dashed lines indicate the first four harmonics of the sample frequency. Both potentiostats show images due to aliasing, without a reconstruction filter. A 4th order low pass filter with a cutoff of 35 kHz eliminates the majority of images in DStat. (green - without filter, red - with filter)
