# Supplementary material for: DStat: A Versatile, Open-Source Potentiostat for Electroanalysis and Integration
Source: PLoS One. 2015 Oct 28;10(10):e0140349. doi: 10.1371/journal.pone.0140349 (PMC4624907; doi:10.1371/journal.pone.0140349)
Supplement: S2 Supporting Information — Discussion of noise properties of current measurement circuit. (PDF) [file pone.0140349.s002.pdf]

S2: Analysis of noise measurements

Fig. 4 in the main text shows a gradual decrease in noise reduction as a function of increasing gain in both experiments (blue diamonds) and simulations (green circles). This can be explained by recognizing two different types of noise sources in the system: voltage noise and current noise. Voltage noise is noise introduced after the current to voltage conversion and is produced by the op-amp of the transimpedance amplifier (U3), as thermal noise in the gain resistor, and as part of the ADC's analogue circuitry. The thermal noise contribution to the voltage noise increases with the square root of the resistance of R_M, while the other components are invariant with gain. Current noise is noise introduced before the transimpedance amplifier's input, including the op-amp's inherent current noise as well as noise coupled into the input line from external sources (e.g., environmental noise from nearby traces on the circuit board). Current noise is amplified by the transimpedance amplifier and its contribution to the output noise is therefore proportional to the gain. The data in Fig. 4 in the main text shows the relationship between the two noise types. At low gain, amplification of current noise is low and voltage noise dominates, resulting in a linear decrease in noise as gain increases. At higher gains, the current noise is amplified significantly and begins to reduce the signal-to-noise ratio enhancement with increasing gain. The practical significance of the noise behaviour is that DStat's signal to noise ratio increases with increasing gain settings throughout the entire range of available measurement resistors and since measurements are never limited by the ADC, the noise response curve can be used to select an appropriate gain for a given signal.

Finally, the observation that the simulation (green circles in Fig. 4 in the main text) indicates slightly lower noise than the experimental data (blue diamonds in Fig. 4 in the main text) can be explained by recognizing that the simulation did not account for noise coupling to the transimpedance amplifier inputs. Thus, the excess noise in the experimental data likely originates from sources beyond the current measurement circuit and could potentially be improved by changes in the circuit board layout, or shielding of the entire DStat.
