# Supplementary material for: DStat: A Versatile, Open-Source Potentiostat for Electroanalysis and Integration
Source: PLoS One. 2015 Oct 28;10(10):e0140349. doi: 10.1371/journal.pone.0140349 (PMC4624907; doi:10.1371/journal.pone.0140349)
Supplement: S3 Supporting Information — Examination of the stability of the potentiostatic circuit under capacitive loads. (PDF) [file pone.0140349.s003.pdf]

# S3: Potentiostatic circuit stability

As with all amplifier circuits, certain operating conditions can cause instability of a potentiostatic circuit which may manifest as overshoot, poorly-damped ringing, or in extreme cases full-scale oscillations at the CE output. Instability occurs when the phase shift in the circuit's feedback loop (the path between the output and the inverting input of U1 in Fig. 2 in the main text) is sufficient to produce positive rather than negative feedback. Many electrochemical cells are especially prone to introducing instability for two reasons: (1) the electrical double layer at the surface of the WE adds a capacitance between the RE and ground, adding phase shift, and (2) extra resistance at the RE increases the phase shift of any capacitance in the feedback loop. The double layer capacitance is proportional to the WE area, with specific capacitances typically ranging between 10 and 40 $\mu F/cm^2$, and is also dependant on the cell potential [1].

While DStat was not designed for large capacitive electrodes, it is capable of operating stably with a purely capacitive load and with well-damped ringing when a 10 kΩ resistance is added at the RE terminal. Stability was assessed by connecting a capacitive load of 1.12 µF between the RE and WE terminals and applying a 1 kHz square wave with an amplitude of 100 mV from DStat's DAC to the potentiostatic circuit. The output of the potentiostatic circuit was measured at the RE terminal with a TDS 2001C oscilloscope (Tektronix, Inc., Oregon, USA). The output with no resistance at the RE terminal (RE and CE shorted) as well as with a RE resistance of 10 kΩ is plotted in Fig. A. The square wave was reproduced without evidence of instability when no resistance was added to the RE and showed some ringing with resistance added to the feedback loop, quickly decaying to the intended output value within 120 µs.

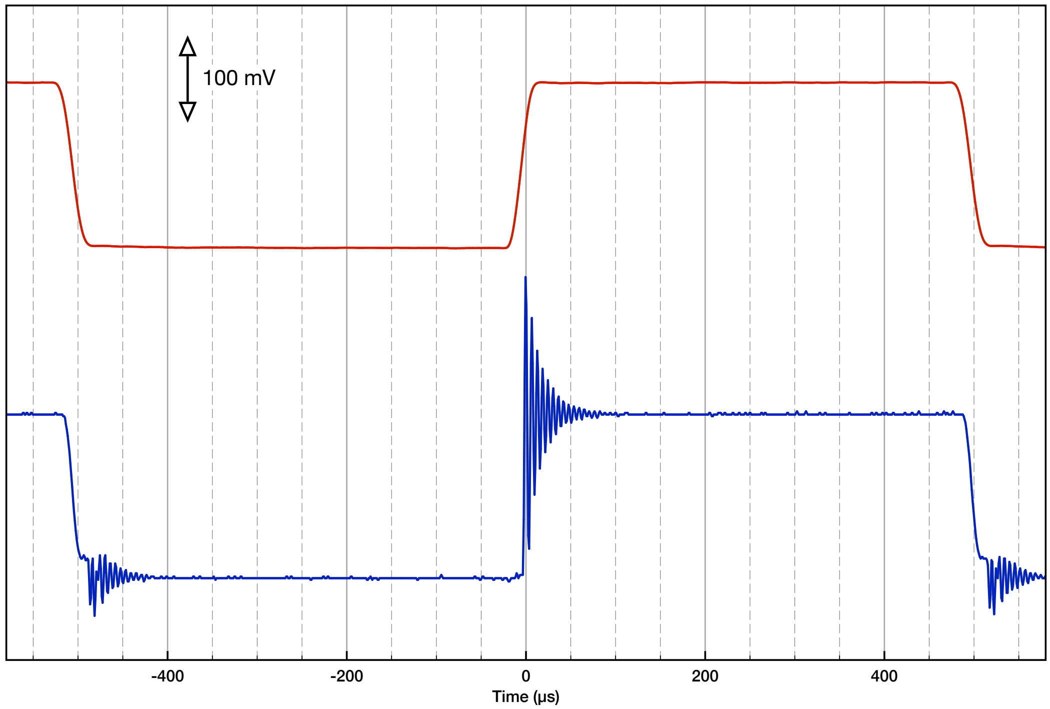


**Figure A. DStat potentiostatic circuit square wave (1 kHz) performance under 1.12 µF capacitive load.** The output measured at the RE terminal with CE and RE terminals shorted is plotted in red (top trace) and shows no signs of instability. The output measured at the RE terminal with a 10 kΩ resistor between CE and RE terminals is plotted in blue (bottom trace) and shows some well-damped ringing, decaying within 120 µs.

## References

1. Bard AJ, Faulkner LR. Electrochemical Methods: Fundamentals and Applications. 2nd ed. Fundamentals and Applications. Wiley; 2000.
