# Supplementary material for: DStat: A Versatile, Open-Source Potentiostat for Electroanalysis and Integration
Source: PLoS One. 2015 Oct 28;10(10):e0140349. doi: 10.1371/journal.pone.0140349 (PMC4624907; doi:10.1371/journal.pone.0140349)
Supplement: S4 Supporting Information — Demonstration of DStat’s potentiometry capabilities by pH measurements and comparison with a commercial pH meter. (PDF) [file pone.0140349.s004.pdf]

S4: Potentiometry

pH 3, 7, and 11 calibration standards were purchased from Fisher Scientific Company (Ottawa ON, CA). pH measurements of each standard were collected using a combination glass pH electrode with integrated Ag/AgCl reference electrode (Beckman Coulter Canada, Mississauga ON, CA) with DStat and an Accumet AR50 benchtop meter (Thermo Fisher Scientific, Waltham MA, USA). The results are shown in Fig. A.

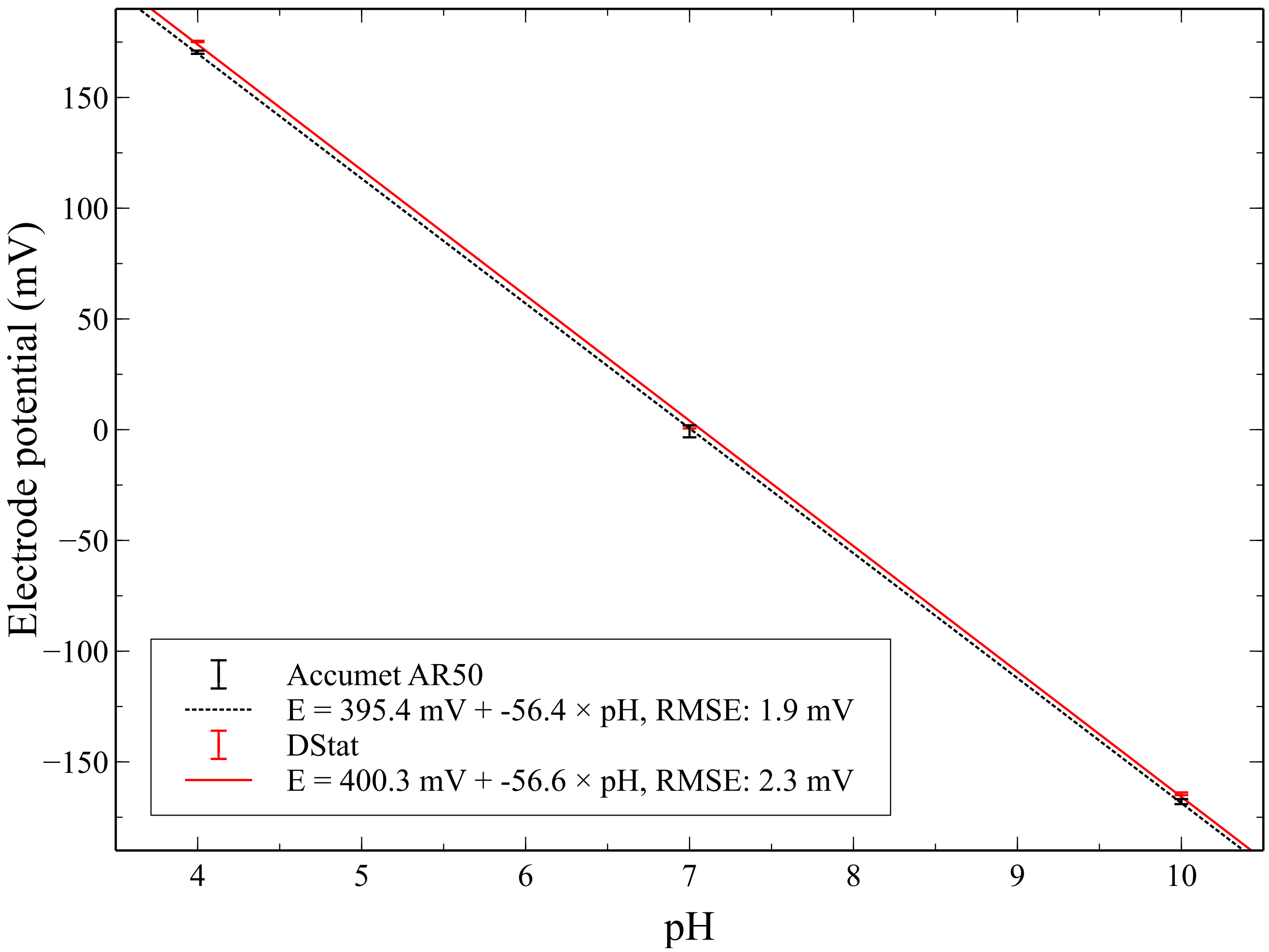

Figure A. Comparison of DStat and Accumet AR50 measurements of pH standard solutions with a combination glass pH electrode. Error bars are ±1 s.d. (n=3). Accumet AR50 measurements are plotted as black bars and DStat measurements as red bars. Linear least squares fit for the Accumet AR50 is shown as a dashed black line and DStat's is shown as a solid red line. RMSE: Root Mean Square Error
