# Supplementary material for: DStat: A Versatile, Open-Source Potentiostat for Electroanalysis and Integration
Source: PLoS One. 2015 Oct 28;10(10):e0140349. doi: 10.1371/journal.pone.0140349 (PMC4624907; doi:10.1371/journal.pone.0140349)
Supplement: S5 Supporting Information — Experimental details of DMF device fabrication and device operation for integration with DStat. (PDF) [file pone.0140349.s005.pdf]

S5: Integration of DStat with Dropbot

DMF device fabrication

Digital microfluidic (DMF) bottom plates were formed with chromium driving electrodes on glass substratesas described previously [1] but with one modification: the use of Fluoropel 1604V (Cytonix LLC, Beltsville, MD, USA) in place of Teflon AF as the hydrophobic coating. Fluoropel 1604V was diluted 1:3 with PFC 110 (Cytonix LLC), to produce a 1% Fluoropel solution. The solution was spin-coated (30 s, 2000 rpm, 500 rpm/s) onto the devices after the Parylene coating step and baked for 10 min at 140 °C. Hybrid DMF top plates bearing electrochemical cells embedded in a DMF-counter-electrode were formed using methods similar to those described by Shamsi et al. [2]. Differences included the use of Fluoropel instead of Teflon-AF for the coating (as above), and the use of a three-electrode cell configuration in place of a two-electrode cell. The cell used here is similar to that described by Dryden et al. [3], comprising a 400 µm-radius circular gold-plated working electrode surrounded by a 30 µm wide annular region containing a counter electrode (270° of annulus) and a silver-plated reference electrode (90° of annulus).

Device operation

Each DMF device was assembled from a bottom plate and a top plate separated by two pieces of 3M Scotch double-sided tape (St. Paul, MN, USA) with a total spacing of approximately 180 µm. Droplet actuation was performed by applying sinusoidal signals of approximately 100 V_{rms} at 10 kHz between the top plate electrodes and electrodes on the bottom plate with the open-source DropBot DMF control system [4]. A 1.5 µL droplet of 10 mM potassium hexacyanoferrate(II) was dispensed from a reservoir and then driven to the electrochemical cell (on the top plate). A cyclic voltammogram (2 scans between -600 mV to 200 mV at 50 mV/s) was acquired with DStat and the droplet was driven away from the electrodes. The interplay between DropBot and DStat is illustrated in Figure 6 in the main text, and video of the whole process is included in S6 Video. (In the video, the recording of the DStat measurement is accelerated 4x for brevity.)

References

1. Ng AHC, Choi K, Luoma RP, Robinson JM, Wheeler AR. Digital Microfluidic Magnetic Separation for Particle-Based Immunoassays. Anal Chem. 2012 Oct;84(20):8805–8812.

2. Shamsi MH, Choi K, Ng AHC, Wheeler AR. A digital microfluidic electrochemical immunoassay. Lab Chip. 2014;14(3):547–554.

3. Dryden MDM, Rackus DDG, Shamsi MH, Wheeler AR. Integrated Digital Microfluidic Platform for Voltammetric Analysis. Anal Chem. 2013;85(18):8809–8816.

4. Fobel R, Fobel C, Wheeler AR. DropBot: An open-source digital microfluidic control system with precise control of electrostatic driving force and instantaneous drop velocity measurement. Appl Phys Lett. 2013;102(19):193513–193518.
