# Supplementary material for: DStat: A Versatile, Open-Source Potentiostat for Electroanalysis and Integration
Source: PLoS One. 2015 Oct 28;10(10):e0140349. doi: 10.1371/journal.pone.0140349 (PMC4624907; doi:10.1371/journal.pone.0140349)
Supplement: S1 File — Electronics manufacturing files, software and firmware source code, and documentation for DStat construction and operation. The most recent version can be retrieved from http://microfluidics.utoronto.ca/dstat. (ZIP) [file pone.0140349.s007.zip › DStat/dstat-hardware.git/dstat-mainboard-pcb.pdf]

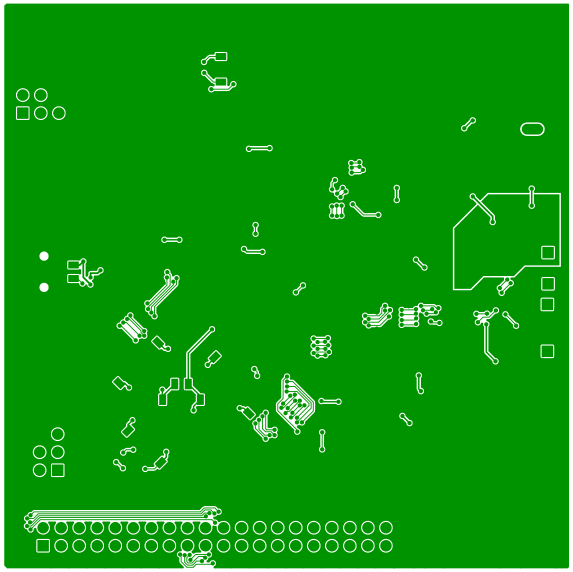

Sheet:		
File:		
**Title:**		
Size: A4	Date:	**Rev:**
KiCad E.D.A. gerbview (2014-09-26 BZR 5151)-product		Id: 1/1

1 2 3 4 5 6

A

B

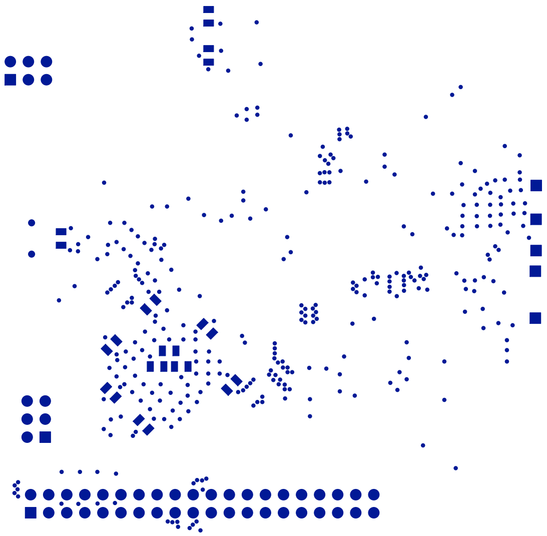

C

D

Sheet:
File:

Title:

Size: A4	Date:	Rev:
KiCad E.D.A. gerbview (2014-09-26 BZR 5151)-product		Id: 1/1

1 2 3 4 5 6

Sheet:
File:

Title:

Size: A4 | Date: | **Rev:**

KiCad E.D.A. gerbview (2014-09-26 BZR 5151)-product | Id: 1/1

Sheet:
File:

Title:

Size: A4	Date:	**Rev:**
KiCad E.D.A. gerbview (2014-09-26 BZR 5151)-product		Id: 1/1

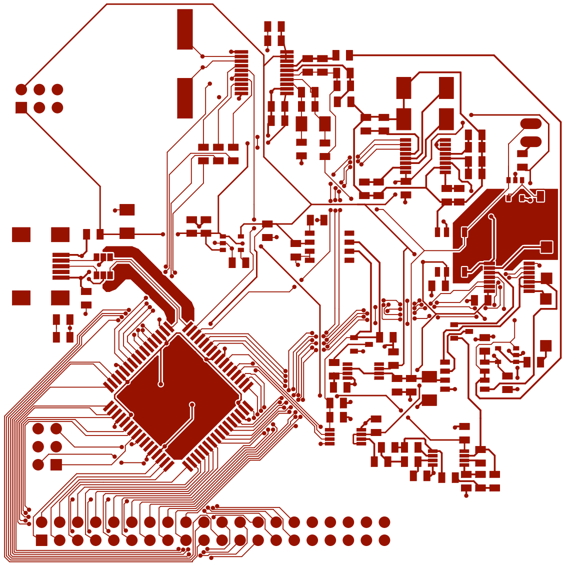

Sheet:		
File:		
Title:		
Size: A4	Date:	**Rev:**
KiCad E.D.A. gerbview (2014-09-26 BZR 5151)-product		Id: 1/1

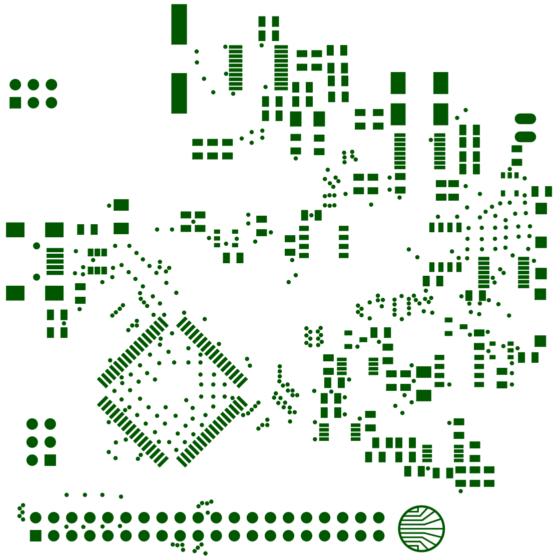

Sheet:
File:

Title:

Size: A4	Date:	**Rev:**
KiCad E.D.A. gerbview (2014-09-26 BZR 5151)-product		Id: 1/1

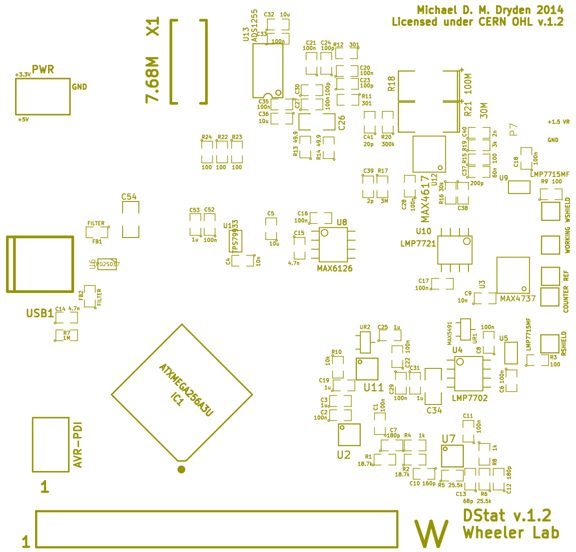

Sheet:
File:
Title:

Size: A4	Date:	**Rev:**
KiCad E.D.A. gerbview (2014-09-26 BZR 5151)-product		Id: 1/1

Sheet:
File:

Title:

Size: A4	Date:	**Rev:**
KiCad E.D.A. gerbview (2014-09-26 BZR 5151)-product		Id: 1/1

Sheet:
File:

Title:

Size: A4 | Date: | **Rev:**

KiCad E.D.A. gerbview (2014-09-26 BZR 5151)-product | Id: 1/1
