# Supplementary material for: DStat: A Versatile, Open-Source Potentiostat for Electroanalysis and Integration
Source: PLoS One. 2015 Oct 28;10(10):e0140349. doi: 10.1371/journal.pone.0140349 (PMC4624907; doi:10.1371/journal.pone.0140349)
Supplement: S1 File — Electronics manufacturing files, software and firmware source code, and documentation for DStat construction and operation. The most recent version can be retrieved from http://microfluidics.utoronto.ca/dstat. (ZIP) [file pone.0140349.s007.zip › DStat/dstat-hardware.git/dstat-mainboard-sch.pdf]

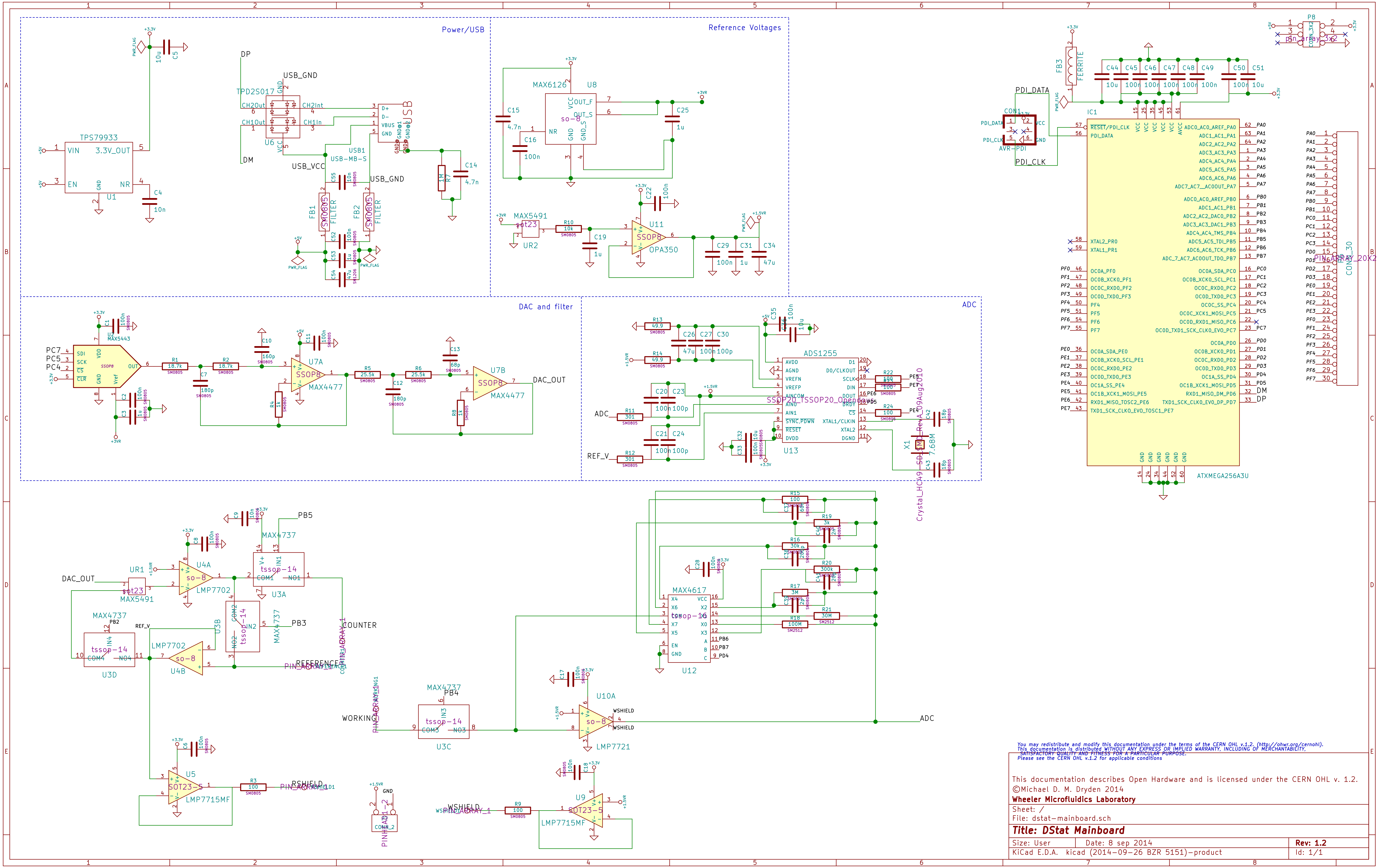

Power/USB
Reference Voltages
DAC and filter
ADC
TPS79933
TPD2S017
USB_GND
USB_VCC
MAX6126
OPA350
MAX5491
MAX5443
MAX4477
ADS1255
ATXMEGA256A3U
MAX4617
MAX4737
LMP7702
LMP7721
LMP7715MF
DAC_OUT
COUNTER
REFERENCE
WORKING
RSHIELD
WSHIELD
PDI_DATA
PDI_CLK
You may redistribute and modify this documentation under the terms of the CERN OHL v.1.2. (http://ohwr.org/cernohl).
This documentation is distributed WITHOUT ANY EXPRESS OR IMPLIED WARRANTY, INCLUDING OF MERCHANTABILITY, SATISFACTORY QUALITY AND FITNESS FOR A PARTICULAR PURPOSE.
Please see the CERN OHL v.1.2 for applicable conditions
This documentation describes Open Hardware and is licensed under the CERN OHL v. 1.2.
©Michael D. M. Dryden 2014
Wheeler Microfluidics Laboratory
Sheet: /
File: dstat-mainboard.sch
Title: DStat Mainboard
Size: User
Date: 8 sep 2014
Rev: 1.2
KiCad E.D.A. kicad (2014-09-26 BZR 5151)-product
Id: 1/1
