# Supplementary material for: DStat: A Versatile, Open-Source Potentiostat for Electroanalysis and Integration
Source: PLoS One. 2015 Oct 28;10(10):e0140349. doi: 10.1371/journal.pone.0140349 (PMC4624907; doi:10.1371/journal.pone.0140349)
Supplement: S1 File — Electronics manufacturing files, software and firmware source code, and documentation for DStat construction and operation. The most recent version can be retrieved from http://microfluidics.utoronto.ca/dstat. (ZIP) [file pone.0140349.s007.zip › DStat/dstat-hardware.git/LICENSE/cern_ohl_v_1_2_howto.pdf]

Guide to the CERN OHL v.1.2

This document contains guidelines on how to apply the CERN OHL v.1.2 to a given hardware design, and on the use of hardware designs licensed under the CERN OHL v.1.2.

How to apply the CERN OHL v.1.2 to a hardware design

Pre-requisite:

Authorship/ownership of the design must be clear and undisputed. Only the legal owner of the rights in the hardware design may decide under what conditions to make it available. If ownership is vested in more than one person/entity, there must be an agreement among the owners to release the hardware design as open hardware, and under the CERN OHL in particular.

The hardware design documentation and files package

Pack all your hardware design documentation files (schematics, layout...) as well as the documents listed below in an archive file. This will ensure the licensee downloads everything in one go. It is best to archive the files using a format everybody can open. Schematics and layouts should be included in both source form and a format readable by everybody, such as pdf.

The following documents must be distributed together with the hardware design documentation:

- Document containing the CERN OHL v.1.2 (e.g. LICENSE.PDF)
- This Guide
- Text files (plain ASCII file), where information can be added to but not removed from, listing:
  - Contact point wishing to receive information about manufactured Products (see section 4.2) (e.g. PRODUCT.TXT);
  - Modifications made by Licensee (see section 3.4.b) (e.g. CHANGES.TXT)

What to do with the hardware design documentation

Include in the hardware design documentation, for instance as a header, the following elements (see section 3.1 CERN OHL v.1.2):

- a copyright notice reflecting actual ownership;
- a notice that the hardware design documentation is licensed under the CERN OHL v.1.2, possibly with a link to http://ohwr.org/cernohl where the licence texts are hosted:
  - "Licensed under CERN OHL v.1.2 or later"
  - "Licensed under CERN OHL v.1.2";
- a disclaimer of warranties;
- a Documentation Location if you wish to specify one;

The following is an example of header if CERN is the Licensor:

> Copyright CERN 2013.
>
> This documentation describes Open Hardware and is licensed under the CERN OHL v. 1.2.
>
> You may redistribute and modify this documentation under the terms of the CERN OHL v.1.2. (http://ohwr.org/cernohl). This documentation is distributed WITHOUT ANY EXPRESS OR IMPLIED WARRANTY, INCLUDING OF MERCHANTABILITY, SATISFACTORY QUALITY AND FITNESS FOR A PARTICULAR PURPOSE. Please see the CERN OHL v.1.2 for applicable conditions

Include in a part of the Documentation corresponding to a visible part of the Product (e.g. silkscreen or top copper for a Printed Circuit Board):

- the licence notice: "Licensed under CERN OHL v.1.2"
  - Do **not** include the CERN logo or the copyright notice
- the Documentation Location if you wish it to appear on the Product, thus enabling all subsequent recipients of the Products to find the Documentation.

How to deal with hardware designs licensed under the CERN OHL v.1.2

Generally speaking, you must always comply with any obligations applying to a particular design (detailed in a contract or accompanying licence). If you receive hardware designs licensed under the CERN OHL v.1.2, the obligations are to:

- Keep intact all the copyright and trademark notices and Documentation Location notices that are on the hardware design documentation;
- Keep intact the references to the CERN OHL v.1.2;
- Keep intact the disclaimer of warranties.

If you modify hardware design that you received from someone else that is licensed under the CERN OHL v.1.2, you must

- Keep intact all the notices referred to above;
- Include notices that you have modified the hardware designs, detailing what modifications where made (e.g. in a CHANGES.TXT file);
- Add the appropriate copyright notice and Documentation Location to the modifications that were made;
- license the modifications under the CERN OHL v.1.2 if you distribute them.
