# Supplementary material for: DStat: A Versatile, Open-Source Potentiostat for Electroanalysis and Integration
Source: PLoS One. 2015 Oct 28;10(10):e0140349. doi: 10.1371/journal.pone.0140349 (PMC4624907; doi:10.1371/journal.pone.0140349)
Supplement: S1 File — Electronics manufacturing files, software and firmware source code, and documentation for DStat construction and operation. The most recent version can be retrieved from http://microfluidics.utoronto.ca/dstat. (ZIP) [file pone.0140349.s007.zip › DStat/dstat-hardware.git/LICENSE/LICENSE.pdf]

CERN Open Hardware Licence v1.2

Preamble

Through this CERN Open Hardware Licence ("CERN OHL") version 1.2, CERN wishes to provide a tool to foster collaboration and sharing among hardware designers.

The CERN OHL is copyright CERN. Anyone is welcome to use the CERN OHL, in unmodified form only, for the distribution of their own Open Hardware designs. Any other right is reserved. Release of hardware designs under the CERN OHL does not constitute an endorsement of the licensor or its designs nor does it imply any involvement by CERN in the development of such designs.

1 Definitions

In this Licence, the following terms have the following meanings:

“Licence” means this CERN OHL.

“Documentation” means schematic diagrams, designs, circuit or circuit board layouts, mechanical drawings, flow charts and descriptive text, and other explanatory material that is explicitly stated as being made available under the conditions of this Licence. The Documentation may be in any medium, including but not limited to computer files and representations on paper, film, or any other media.

“Documentation Location” means a location where the Licensor has placed Documentation, and which he believes will be publicly accessible for at least three years from the first communication to the public or distribution of Documentation.

“Product” means either an entire, or any part of a, device built using the Documentation or the modified Documentation.

“Licensee” means any natural or legal person exercising rights under this Licence.

“Licensor” means any natural or legal person that creates or modifies Documentation and subsequently communicates to the public and/ or distributes the resulting Documentation under the terms and conditions of this Licence.

A Licensee may at the same time be a Licensor, and vice versa.

Use of the masculine gender includes the feminine and neuter genders and is employed solely to facilitate reading.

2 Applicability

2.1 This Licence governs the use, copying, modification, communication to the public and distribution of the Documentation, and the manufacture and distribution of Products. By exercising any right granted under this Licence, the Licensee irrevocably accepts these terms and conditions.

2.2 This Licence is granted by the Licensor directly to the Licensee, and shall apply worldwide and without limitation in time. The Licensee may assign his licence rights or grant sub-licences.

2.3 This Licence does not extend to software, firmware, or code loaded into programmable devices which may be used in conjunction with the Documentation, the modified Documentation or with Products, unless such software, firmware, or code is explicitly expressed to be subject to this Licence. The use of such software, firmware, or code is otherwise subject to the applicable licence terms and conditions.

3 Copying, modification, communication to the public and distribution of the Documentation

3.1 The Licensee shall keep intact all copyright and trademarks notices, all notices referring to Documentation Location, and all notices that refer to this Licence and to the disclaimer of warranties that are included in the Documentation. He shall include a copy thereof in every copy of the Documentation or, as the case may be, modified Documentation, that he communicates to the public or distributes.

3.2 The Licensee may copy, communicate to the public and distribute verbatim copies of the Documentation, in any medium, subject to the requirements specified in section 3.1.

3.3 The Licensee may modify the Documentation or any portion thereof provided that upon modification of the Documentation, the Licensee shall make the modified Documentation available from a Documentation Location such that it can be easily located by an original Licensor once the Licensee communicates to the public or distributes the modified Documentation under section 3.4, and, where required by section 4.1, by a recipient of a Product. However, the Licensor shall not assert his rights under the foregoing proviso unless or until a Product is distributed.

3.4 The Licensee may communicate to the public and distribute the modified Documentation (thereby in addition to being a Licensee also becoming a Licensor), always provided that he shall:

a) comply with section 3.1;

b) cause the modified Documentation to carry prominent notices stating that the Licensee has modified the Documentation, with the date and description of the modifications;

c) cause the modified Documentation to carry a new Documentation Location notice if the original Documentation provided for one;

d) make available the modified Documentation at the same level of abstraction as that of the Documentation, in the preferred format for making modifications to it (e.g. the native format of the CAD tool as applicable), and in the event that format is proprietary, in a format viewable with a tool licensed under an OSI-approved license if the proprietary tool can create it; and

e) license the modified Documentation under the terms and conditions of this Licence or, where applicable, a later version of this Licence as may be issued by CERN.

3.5 The Licence includes a non-exclusive licence to those patents or registered designs that are held by, under the control of, or sub-licensable by the Licensor, to the extent necessary to make use of the rights granted under this Licence. The scope of this section 3.5 shall be strictly limited to the parts of the Documentation or modified Documentation created by the Licensor.

4 Manufacture and distribution of Products

4.1 The Licensee may manufacture or distribute Products always provided that, where such manufacture or distribution requires a licence under this Licence the Licensee provides to each recipient of such Products an easy means of accessing a copy of the Documentation or modified Documentation, as applicable, as set out in section 3.

4.2 The Licensee is invited to inform any Licensor who has indicated his wish to receive this information about the type, quantity and dates of production of Products the Licensee has (had) manufactured

5 Warranty and liability

5.1 DISCLAIMER – The Documentation and any modified Documentation are provided "as is" and any express or implied warranties, including, but not limited to, implied warranties of merchantability, of satisfactory quality, non-infringement of third party rights, and fitness for a particular purpose or use are disclaimed in respect of the Documentation, the modified Documentation or any Product. The Licensor makes no representation that the Documentation, modified Documentation, or any Product, does or will not infringe any patent, copyright, trade secret or other proprietary right. The entire risk as to the use, quality, and performance of a Product shall be with the Licensee and not the Licensor. This disclaimer of warranty is an

essential part of this Licence and a condition for the grant of any rights granted under this Licence. The Licensee warrants that it does not act in a consumer capacity.

5.2 LIMITATION OF LIABILITY – The Licensor shall have no liability for direct, indirect, special, incidental, consequential, exemplary, punitive or other damages of any character including, without limitation, procurement of substitute goods or services, loss of use, data or profits, or business interruption, however caused and on any theory of contract, warranty, tort (including negligence), product liability or otherwise, arising in any way in relation to the Documentation, modified Documentation and/or the use, manufacture or distribution of a Product, even if advised of the possibility of such damages, and the Licensee shall hold the Licensor(s) free and harmless from any liability, costs, damages, fees and expenses, including claims by third parties, in relation to such use.

6 General

6.1 Except for the rights explicitly granted hereunder, this Licence does not imply or represent any transfer or assignment of intellectual property rights to the Licensee.

6.2 The Licensee shall not use or make reference to any of the names (including acronyms and abbreviations), images, or logos under which the Licensor is known, save in so far as required to comply with section 3. Any such permitted use or reference shall be factual and shall in no event suggest any kind of endorsement by the Licensor or its personnel of the modified Documentation or any Product, or any kind of implication by the Licensor or its personnel in the preparation of the modified Documentation or Product.

6.3 CERN may publish updated versions of this Licence which retain the same general provisions as this version, but differ in detail so far this is required and reasonable. New versions will be published with a unique version number.

6.4 This Licence shall terminate with immediate effect, upon written notice and without involvement of a court if the Licensee fails to comply with any of its terms and conditions, or if the Licensee initiates legal action against Licensor in relation to this Licence. Section 5 shall continue to apply.
