# Supplementary material for: DStat: A Versatile, Open-Source Potentiostat for Electroanalysis and Integration
Source: PLoS One. 2015 Oct 28;10(10):e0140349. doi: 10.1371/journal.pone.0140349 (PMC4624907; doi:10.1371/journal.pone.0140349)
Supplement: S1 File — Electronics manufacturing files, software and firmware source code, and documentation for DStat construction and operation. The most recent version can be retrieved from http://microfluidics.utoronto.ca/dstat. (ZIP) [file pone.0140349.s007.zip › DStat/dstat-interface.git/images/k.pdf]

Master Card Details

5191-[illegible]

Current Balance does not include pending interest charges or transactions that have been authorized but not yet posted to your account.

If you see a charge that does not match your records please let us know. Disputed items must be reported within 30 days of the statement date.

MasterCard Details
Credit Limit:
Available Credit: (May 21)

May 21, 2015 Statement
View Details
Statement Balance:
Minimum Payment:

Reward Program: AIR MILES
[illegible]

Transactions

Use the drop-down menus below to view transactions since your last statement, view one of your last three statements or to sort your transactions by account access if applicable. Select Download Account Details to download up to three months worth of transactions. Select Request a Statement Copy to order paper copies of statements older than the most recent three.

May 21, 2015 | Account Access

Transaction Date	Posting Date	Description	Amount
**Card Number: 5191-**			
[illegible]			
[illegible]			
[illegible]			
[illegible]			
[illegible]			
[illegible]			
[illegible]			
[illegible]			
[illegible]			
[illegible]			
[illegible]			
[illegible]			
[illegible]			
[illegible]			
12/05/2015	13/05/2015	COLLEGE HOME HARDWARE TORONTO ON	$35.79

14/05/2015	15/05/2015	DAVIDSON'S VALU MART # TORONTO ON	$21.98
15/05/2015	18/05/2015	METRO #742 TORONTO ON	$27.07
19/05/2015	20/05/2015	Amazon.ca AMAZON.CA ON	$45.94
19/05/2015	20/05/2015	DAVIDSON'S VALU MART # TORONTO ON	$9.63
20/05/2015	21/05/2015	APL* ITUNES.COM/BILL 800-676-2775 ON	$1.29

View additional account details on your monthly statement

Report any items which do not agree with your records within 30 days of statement date.

Your payment is due on the due date and is subject to the terms and conditions within the Cardholder Agreement.
