# Supplementary figures and images for: DStat: A Versatile, Open-Source Potentiostat for Electroanalysis and Integration
Source: PLoS One. 2015 Oct 28;10(10):e0140349. doi: 10.1371/journal.pone.0140349 (PMC4624907; doi:10.1371/journal.pone.0140349)

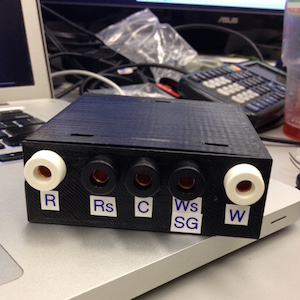

Supplement: S1 File — Electronics manufacturing files, software and firmware source code, and documentation for DStat construction and operation. The most recent version can be retrieved from http://microfluidics.utoronto.ca/dstat. (ZIP) [file pone.0140349.s007.zip › DStat/dstat-hardware.git/images/dstat1.jpg]

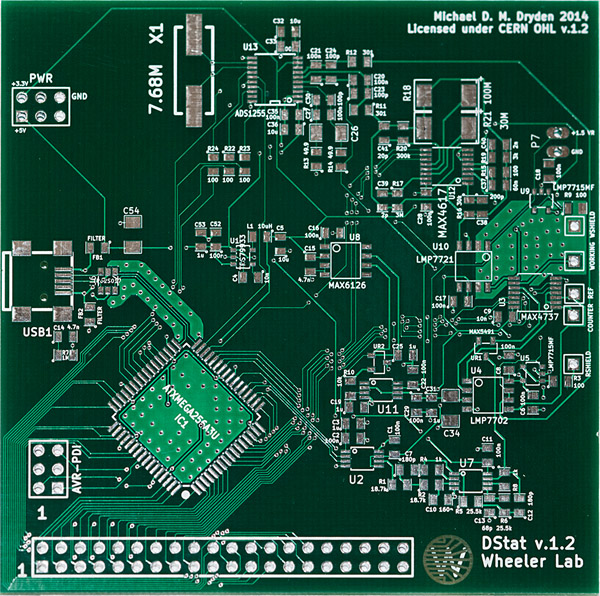

Supplement: S1 File — Electronics manufacturing files, software and firmware source code, and documentation for DStat construction and operation. The most recent version can be retrieved from http://microfluidics.utoronto.ca/dstat. (ZIP) [file pone.0140349.s007.zip › DStat/dstat-hardware.git/images/Full_PCB.jpg]

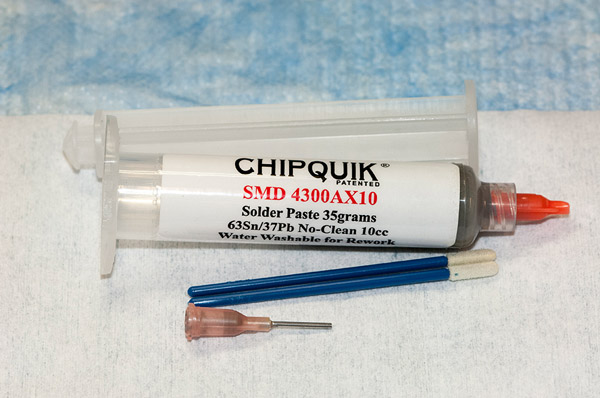

Supplement: S1 File — Electronics manufacturing files, software and firmware source code, and documentation for DStat construction and operation. The most recent version can be retrieved from http://microfluidics.utoronto.ca/dstat. (ZIP) [file pone.0140349.s007.zip › DStat/dstat-hardware.git/images/paste.jpg]

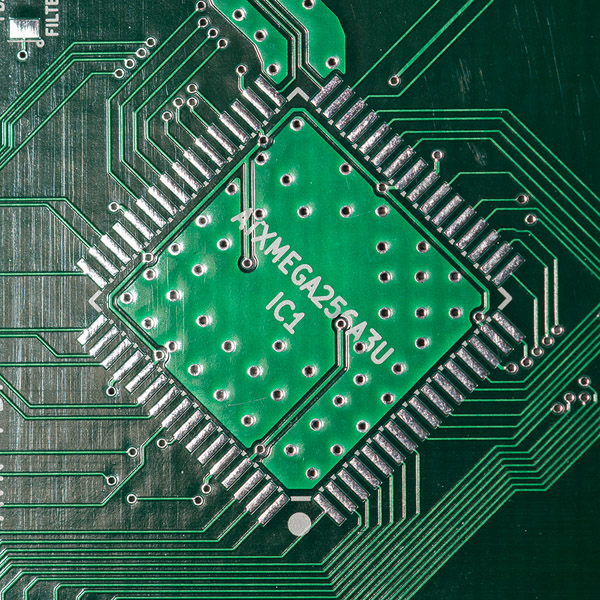

Supplement: S1 File — Electronics manufacturing files, software and firmware source code, and documentation for DStat construction and operation. The most recent version can be retrieved from http://microfluidics.utoronto.ca/dstat. (ZIP) [file pone.0140349.s007.zip › DStat/dstat-hardware.git/images/PCB-bare.jpg]

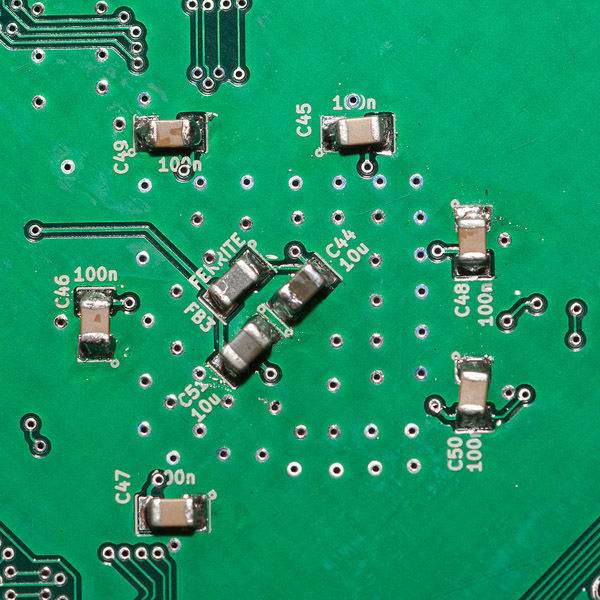

Supplement: S1 File — Electronics manufacturing files, software and firmware source code, and documentation for DStat construction and operation. The most recent version can be retrieved from http://microfluidics.utoronto.ca/dstat. (ZIP) [file pone.0140349.s007.zip › DStat/dstat-hardware.git/images/PCB_back.jpg]

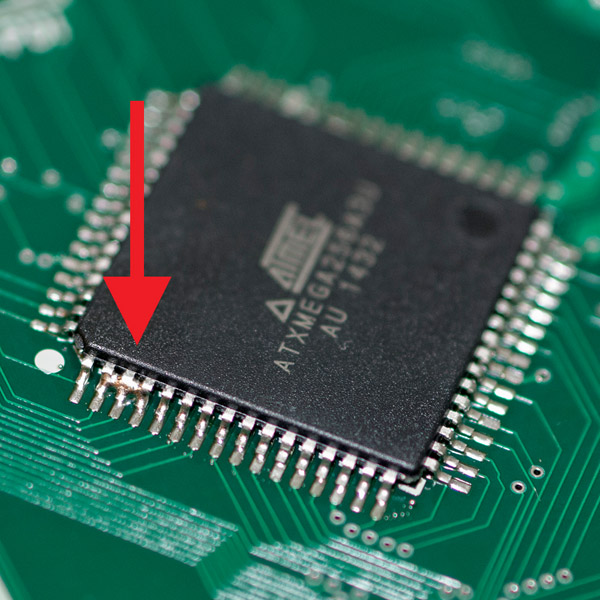

Supplement: S1 File — Electronics manufacturing files, software and firmware source code, and documentation for DStat construction and operation. The most recent version can be retrieved from http://microfluidics.utoronto.ca/dstat. (ZIP) [file pone.0140349.s007.zip › DStat/dstat-hardware.git/images/PCB_bridged_pins.jpg]

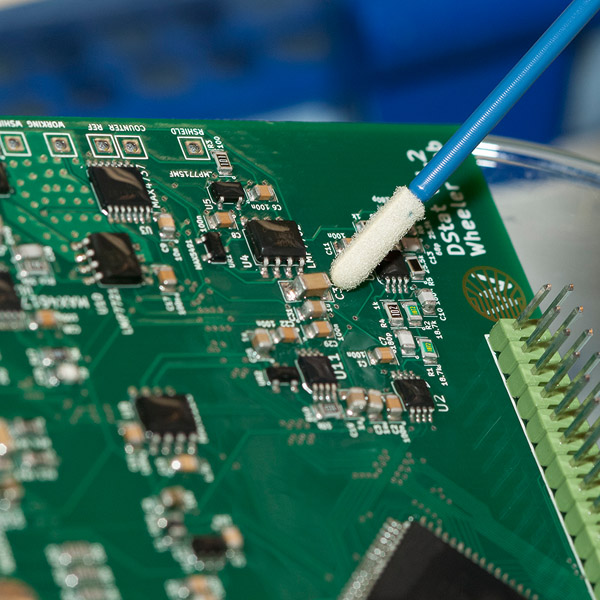

Supplement: S1 File — Electronics manufacturing files, software and firmware source code, and documentation for DStat construction and operation. The most recent version can be retrieved from http://microfluidics.utoronto.ca/dstat. (ZIP) [file pone.0140349.s007.zip › DStat/dstat-hardware.git/images/PCB_clean.jpg]

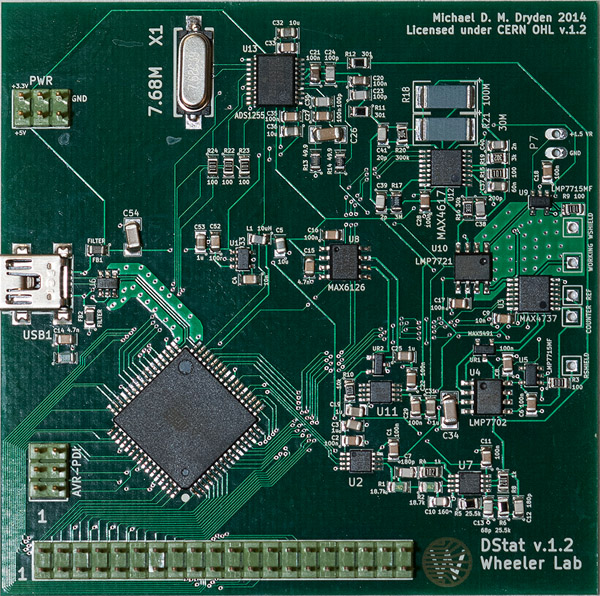

Supplement: S1 File — Electronics manufacturing files, software and firmware source code, and documentation for DStat construction and operation. The most recent version can be retrieved from http://microfluidics.utoronto.ca/dstat. (ZIP) [file pone.0140349.s007.zip › DStat/dstat-hardware.git/images/PCB_finished.jpg]

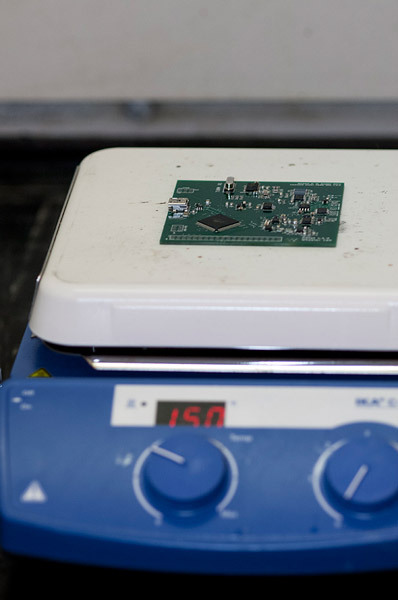

Supplement: S1 File — Electronics manufacturing files, software and firmware source code, and documentation for DStat construction and operation. The most recent version can be retrieved from http://microfluidics.utoronto.ca/dstat. (ZIP) [file pone.0140349.s007.zip › DStat/dstat-hardware.git/images/PCB_hotplate.jpg]

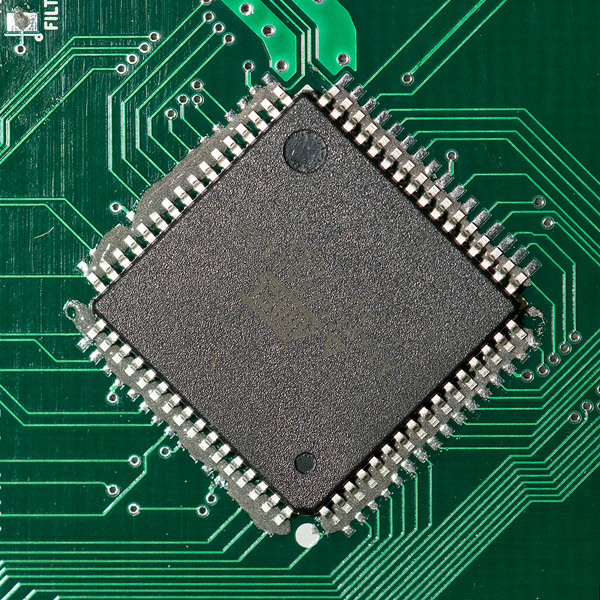

Supplement: S1 File — Electronics manufacturing files, software and firmware source code, and documentation for DStat construction and operation. The most recent version can be retrieved from http://microfluidics.utoronto.ca/dstat. (ZIP) [file pone.0140349.s007.zip › DStat/dstat-hardware.git/images/PCB_parts.jpg]

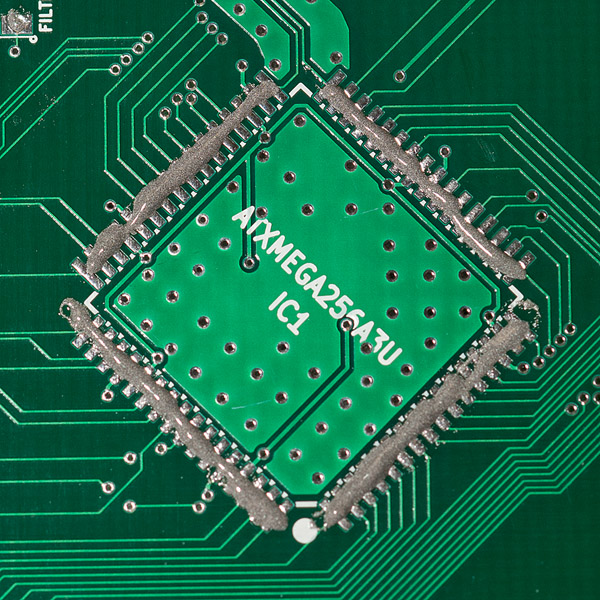

Supplement: S1 File — Electronics manufacturing files, software and firmware source code, and documentation for DStat construction and operation. The most recent version can be retrieved from http://microfluidics.utoronto.ca/dstat. (ZIP) [file pone.0140349.s007.zip › DStat/dstat-hardware.git/images/PCB_paste1.jpg]

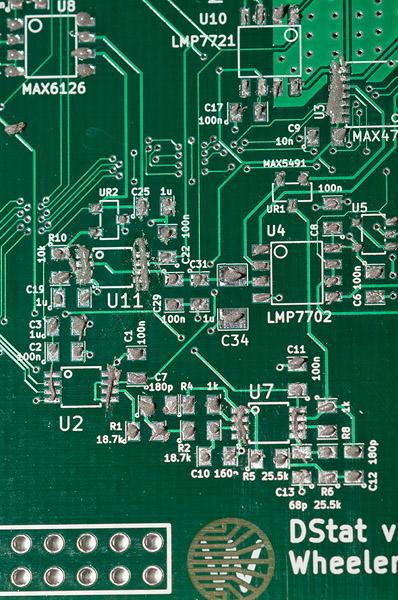

Supplement: S1 File — Electronics manufacturing files, software and firmware source code, and documentation for DStat construction and operation. The most recent version can be retrieved from http://microfluidics.utoronto.ca/dstat. (ZIP) [file pone.0140349.s007.zip › DStat/dstat-hardware.git/images/PCB_paste2.jpg]

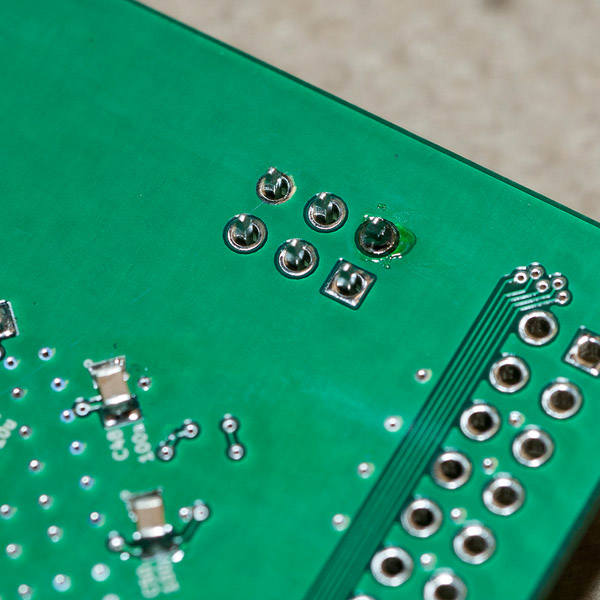

Supplement: S1 File — Electronics manufacturing files, software and firmware source code, and documentation for DStat construction and operation. The most recent version can be retrieved from http://microfluidics.utoronto.ca/dstat. (ZIP) [file pone.0140349.s007.zip › DStat/dstat-hardware.git/images/PCB_through_hole.jpg]

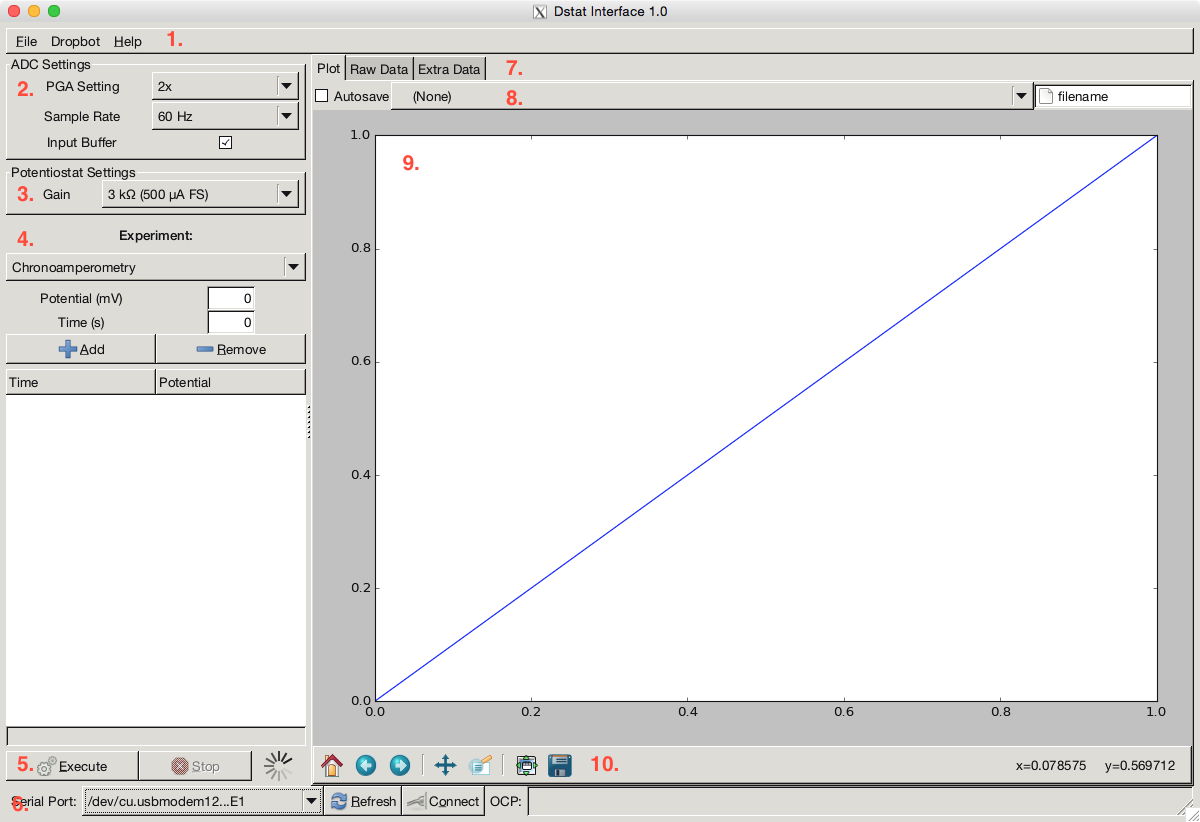

Supplement: S1 File — Electronics manufacturing files, software and firmware source code, and documentation for DStat construction and operation. The most recent version can be retrieved from http://microfluidics.utoronto.ca/dstat. (ZIP) [file pone.0140349.s007.zip › DStat/dstat-interface.git/images/1.png]

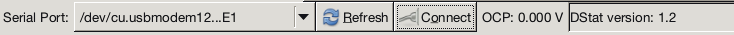

Supplement: S1 File — Electronics manufacturing files, software and firmware source code, and documentation for DStat construction and operation. The most recent version can be retrieved from http://microfluidics.utoronto.ca/dstat. (ZIP) [file pone.0140349.s007.zip › DStat/dstat-interface.git/images/2.png]

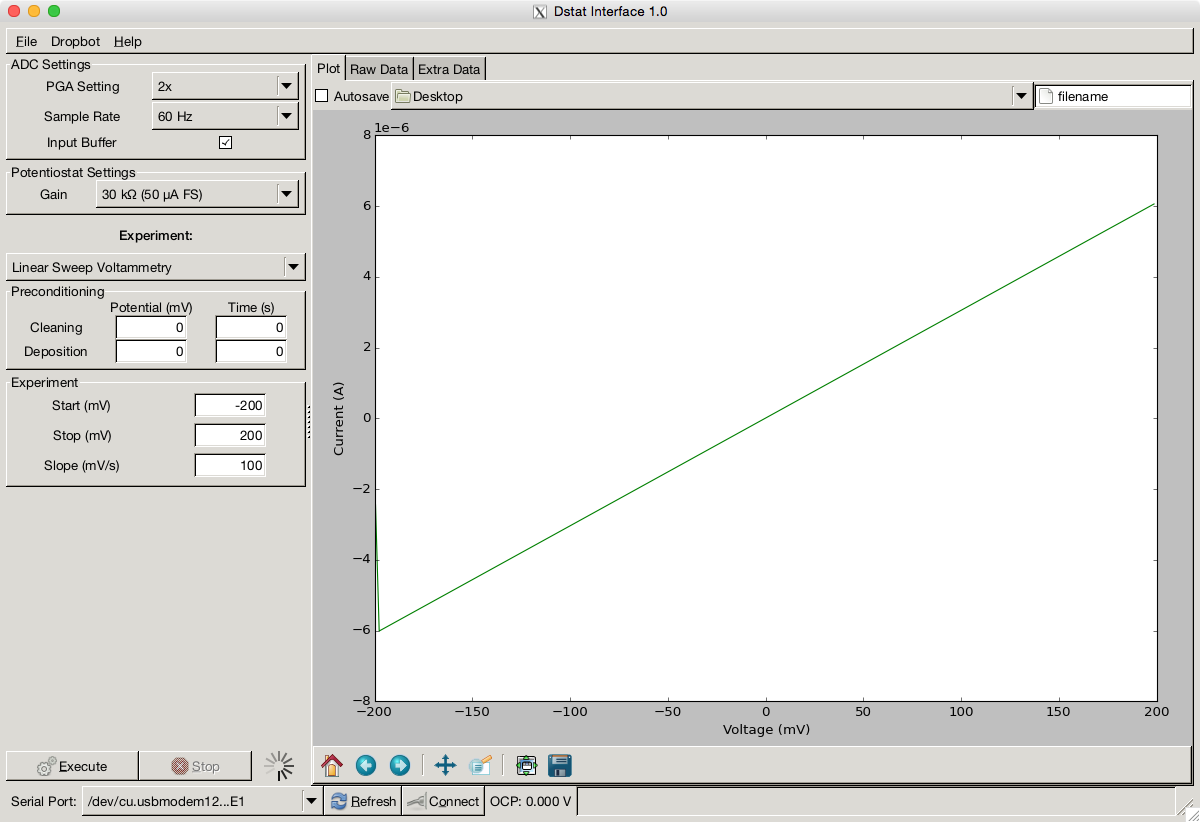

Supplement: S1 File — Electronics manufacturing files, software and firmware source code, and documentation for DStat construction and operation. The most recent version can be retrieved from http://microfluidics.utoronto.ca/dstat. (ZIP) [file pone.0140349.s007.zip › DStat/dstat-interface.git/images/3.png]

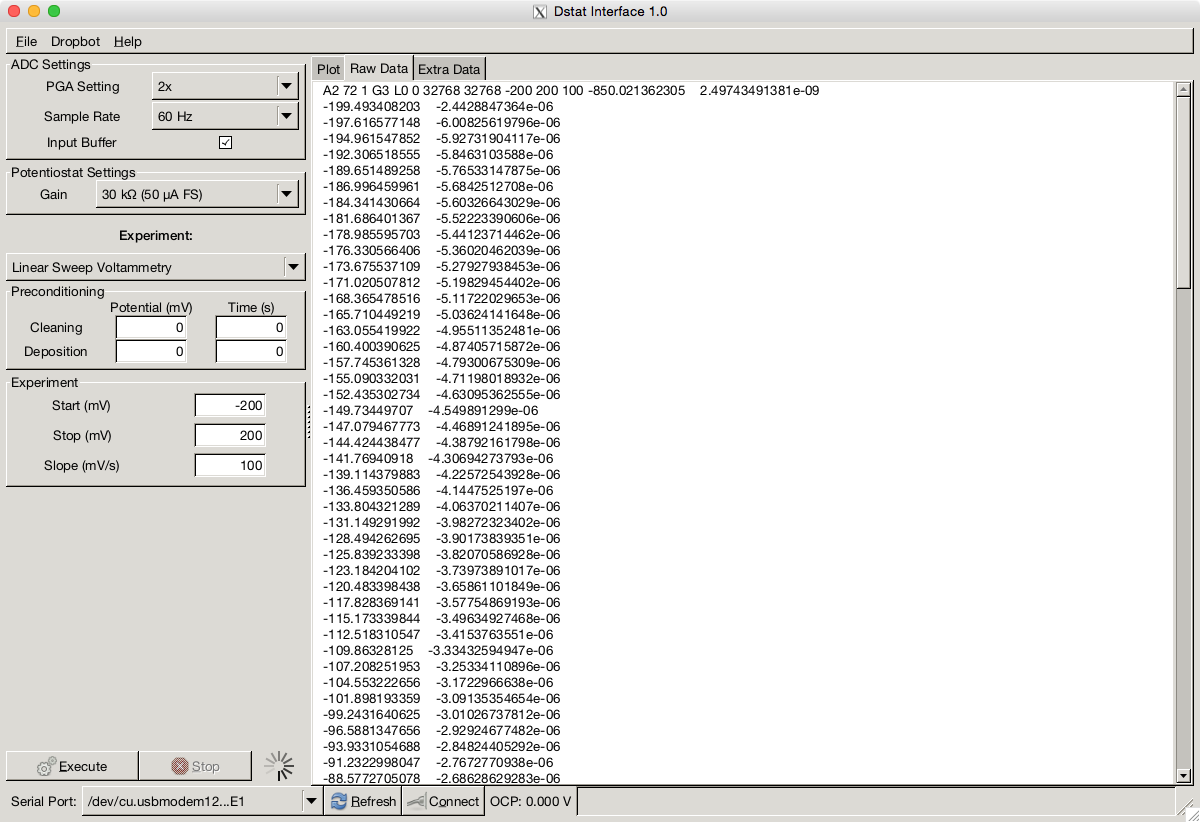

Supplement: S1 File — Electronics manufacturing files, software and firmware source code, and documentation for DStat construction and operation. The most recent version can be retrieved from http://microfluidics.utoronto.ca/dstat. (ZIP) [file pone.0140349.s007.zip › DStat/dstat-interface.git/images/4.png]

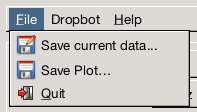

Supplement: S1 File — Electronics manufacturing files, software and firmware source code, and documentation for DStat construction and operation. The most recent version can be retrieved from http://microfluidics.utoronto.ca/dstat. (ZIP) [file pone.0140349.s007.zip › DStat/dstat-interface.git/images/5.png]

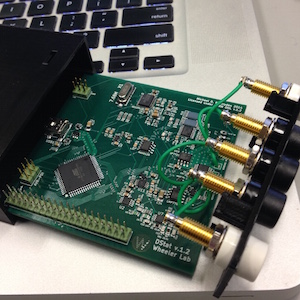

Supplement: S1 File — Electronics manufacturing files, software and firmware source code, and documentation for DStat construction and operation. The most recent version can be retrieved from http://microfluidics.utoronto.ca/dstat. (ZIP) [file pone.0140349.s007.zip › DStat/dstat2.jpg]
